# Solvent-Free Ultrasonic Dispersion of Nanofillers in Epoxy Matrix

**DOI:** 10.3390/polym13020308

**Published:** 2021-01-19

**Authors:** Benjamin Zanghellini, Patrick Knaack, Sebastian Schörpf, Karl-Heinz Semlitsch, Helga C. Lichtenegger, Bernhard Praher, Maria Omastova, Harald Rennhofer

**Affiliations:** 1 Instiute of Physics and Materials Science, University of Natural Resources and Life Sciences Vienna, 1190 Vienna, Austria; helga.lichtenegger@boku.ac.at (H.C.L.); harald.rennhofer@boku.ac.at (H.R.); 2 Instiute of Applied Synthetic Chemistry, Technical University of Vienna, 1190 Vienna, Austria; patrick.knaack@tuwien.ac.at (P.K.); sebastian.schoerpf@tuwien.ac.at (S.S.); 3 Secar Technology GmbH, 8682 Mürzzuschlag, Austria; kh.semlitsch@secar.at; 4 Institute of Polymer Injection Moulding and Process Automation, Johannes Kepler University Linz, 4040 Linz, Austria; bernhard.praher@jku.at; 5 Polymer Institute, Slovak Academy of Sciences, Dubravska cesta 9, 845 41 Bratislava, Slovakia; maria.omastova@savba.sk

**Keywords:** ultrasonication, dispersion, functionalization, carbon nanotubes, rheology

## Abstract

Dispersion of carbon nanotubes and carbon nanofibers is a crucial processing step in the production of polymer-based nanocomposites and poses a great challenge due to the tendency of nanofillers to agglomerate. One of the most effective methods for dispersion is the use of a three-roll mill, which is a well-established method and results in agglomerates below 5 µm. Nevertheless, this process is time-consuming and thus a limiting factor for industrial applications. Our aim was to establish an easy and efficient ultrasonic dispersion process, characterize the dispersion parameters, and compare both methods, ultrasonication and the three-roll mill. We applied rheological tests and analyzed the agglomerate sizes by an image fit of the microscopy images. All these analyses combined deliver a valuable set of information about the dispersion’s quality and, therefore, allows the improvement and further adaptation of the dispersion process.

## 1. Introduction

Carbon nanotubes (CNT) or carbon nanofibers (CNF) used as nanofillers gain more and more interest as reinforcement materials in the field of polymer-based composite materials due to their outstanding mechanical, electronic, thermal, and optical properties in combination with a unique aspect ratio and surface to volume ratio [1,2,3,4]. In fiber reinforced polymers, the matrix mainly transfers and distributes external forces to the fibers. Thus, a sufficient bonding between the polymer and the fiber is crucial for an effective load transfer [5,6]. It is widely known that a majority of failures arise when the composites experience loadings off the main strain axis due to intra- or interlaminar delamination [7]. The addition of nanofillers to the polymer can boost the fiber-matrix bonding and strengthen the resistance against mechanical failure of the reinforced composite. Nevertheless, the stress transmission is limited and dependent on the polymer-nanofiller interface [8], since CNT also tend to interact, re-agglomerate, and lock even in dispersion due to van der Waals forces and their high aspect ratio [9]. Functionalization by a chemical modification of the CNT surface helps to further enhance the binding to the epoxy matrix and can be achieved by several methods [10,11,12]. The introduced functional groups can then react with the epoxy resin similar to the polymer-hardener reaction and strengthen the interface binding [13]. At least as important is the dispersion of the nanofillers in the matrix. Over the years, several methods were established to achieve this task [14]. To mechanically break up agglomerates, an applied shear stress must be bigger than the agglomerate strength; for lower shear forces, the dispersion is mainly based on erosion. Two widely used methods fulfilling this task properly are calendaring on a three-roll mill (TRM) directly in the resin [15] and ultrasonication (US) in an additional solvent system [9]. TRM appeared to be one of the most effective methods to properly disperse nanofillers [16,17]. In this conventional route, the nanofillers are dispersed in the epoxy via calendaring, and in the next step, the dispersion is mixed with the hardener for curing. This process is generally very time consuming and thus not very suitable for industrial use [12]. Considering dispersing via US, one has to take into account that long dispersion times and high induced power of the US system can lead to a shortening of the nanofillers in length during the dispersion process [18,19,20]. This leads to an altered aspect ratio, which influences the dispersibility of the nanofiller. To check on such reduction, several methods can be applied: Sesis et al. used atomic force microscopy [18], Wu et al. used scanning electron microscopy in combination with Fourier Transform Infrared spectroscopy [19], or transmission electron microscopy [20]. Further, US has one main disadvantage: due to the high viscosity of epoxy resin systems, dispersions are only accomplished using additional solvents. Dehghan et al. reported on dispersing 1–3 wt% multi-walled carbon nanotubes (MWCNT) in a bisphenol-A epoxy resin by using acetone as a solvent system. To get rid of the acetone in the final dispersion, the mixture had to be heated at 65 °C for 1 h [21]. In another study, p-xylene and dichloromethane were used as solvents to disperse MWCNT with filler grades from 1 to 3 wt% in polycarbonate [22]. Rezazadeh et al. ultrasonically dispersed MWCNT first in xylene and an additional dispersing agent (Disperbyk 163) and added the epoxy resin through shear mixing of 800 rpm for 60 min. The solvent was removed under vacuum (<200 mbar) [23]. Fromyr et al. dispersed 1 wt% MWCNT directly in hardener using 3 wt% Disperbyk 2150 as additional dispersing agent and used dynamic light scattering to estimate the particle size distributions. This method is restricted by the fact that particle sizes larger than 2 µm cannot be detected [24]. Little is reported on incorporation methods by directly dispersing the nanotubes in the resin-hardener mixture [25,26]. In both studies, the dispersion quality was not investigated. These examples already show that most of the additional solvents used are noxious and thus excessive use should be avoided. Additionally, as a common aspect of all these studies, it can be observed that the dispersion quality is not reported in detail or investigated at all. Nevertheless, the dispersion quality can be analyzed by different means, e.g., microscopy and rheology. Microscopical analysis can be performed in combination with image processing tools to determine particle size distributions. For a quantitative agglomerate size analysis, the determination of the Feret diameter [27] can be used, which was successfully applied in other studies before [28,29]. While microscopy gives direct information that is anyhow limited to bigger length scales (micrometer regime), rheology allows to access integral parameters like viscoelastic character, long-term stability, and sedimentation.

The interpretation of those rheological properties is well documented in literature [30,31,32]. Wu et al. found that MWCNT reduce the linear viscoelastic region of dispersions, especially for higher filler grades. In addition to that, an increase of viscosity values and the storage modulus G’ due to the presence of nanofillers can be attributed to a formed percolation network; it is even more pronounced for increasing filler grades. The formed network can further be identified by the ratio G’:G’’ of storage and loss modulus, where values >1 indicate a percolated system [33]. The slopes of G’ and G’’ are decreasing with increasing filler grades and can be explained by stronger polymer-nanotube or nanotube-nanotube interactions and, again, the formation of a network [34]. For reinforced dispersions, frequency-independent behavior of the storage modulus G’ is reported at lower frequencies (in an applied frequency sweep test), indicating strong polymer-particle interactions [35]. MWCNT also showed strong shear thinning behavior in dispersion; this effect was even more pronounced in surface modified MWCNT (acid, plasma, or amine treated MWCNT). Kim et al. reasoned this by the interfacial bonding between CNT and the polymer, which is more pronounced with modified nanofillers [36].

In this study, we aim at a new and straightforward approach to produce proper dispersions in a time-saving process: carbon nanotubes, carbon nanofibers, and functionalized carbon nanotubes are dispersed in a tetrahydromethylphthalic anhydride hardener matrix system through direct ultrasonication without the use of any additional solvents. For reference, samples with the same filler grades are produced via calendaring on a TRM. The dispersion quality of both methods is analyzed and compared quantitatively. Microscopic images were recorded and analyzed on agglomerate sizes and size distribution. Various rheological tests were applied to study the dispersion quality by different rheological parameters.

## 2. Materials and Methods

### 2.1. Matrix and Nanofillers

In the course of this study, we used a bisphenol-A-epichlorhydrin resin (Biresin CR141, Sika GmbH, Stuttgart, Germany) in combination with a tetrahydromethylphthalic anhydride hardener (Biresin CH141, Sika GmbH, Stuttgart, Germany). The mixing ratio of the two components is 10:9 by weight.

Three different nanofillers were used in this study: multi-wall carbon nanotubes (CNT) from Nanocyl (NC7000, Nanocyl, S.A., Sambreville, Belgium), NC7000 oxidized in-house, and carbon nanofibers from Sigma Aldrich (Sigma Aldrich, Vienna, Austria). According to the supplier, the CNTs were produced via Catalytic Chemical Vapor Deposition (CCVD) and have an average diameter of 9.5 nm and an average length of 1.5 µm with a purity of 90% carbon determined via thermogravimetric analysis (TGA). The CNF were graphitized (iron-free) conical platelets with sizes of 100 nm × 20–200 µm. Fillers are denoted CNT, CNToxi, and CNF throughout the manuscript. Filler amounts of 0.5 wt%, 1.0 wt%, and 1.5 wt% with respect to the dispersion medium were chosen, resulting in filler contents of 0.25 wt%, 0.5 wt%, and 0.75 wt% in the composite. These filler contents were chosen based on previous results [12,37] and reports, that even filler amounts of 0.1 wt% lead to enhanced mechanical properties [38]. Preliminary tests also showed considerably longer dispersion times and agglomeration for a higher amount of filler. To qualitatively show the success of the dispersion methods, we produced a negative control: 0.5 wt% CNT were additionally dispersed in a non-optimal way. These dispersions are denoted further on as semi-dispersed.

### 2.2. Oxidation

Carbon nanotubes tend, even in dispersion, to re-agglomerate due to their size to aspect ratio and due to van der Waals or Coulomb forces. Oxidation of CNTs can counteract those effects. Previously, we established an eco-friendly method to oxidate CNTs [12]. This could be further improved by functionalizing the nanotubes with 30% hydrogen peroxide at room temperature (21 °C), only on a magnetic stirrer in a constant stirring process for 7 days. To check, if the oxidation process was successful, samples (reference, oxidation at room temperature, oxidation at elevated temperature) were tested via X-ray photoelectron spectroscopy (XPS). The degree of oxidation achieved with the new method was comparable to our previous results. All CNToxi used for the experiments in this manuscript were produced by the room temperature method in one single batch.

### 2.3. Dispersion

Nanofiller were dispersed by two different routes—TRM and US—to clarify to what extent the dispersion quality of both methods is comparable, and if a satisfactory dispersion quality of ultrasonication preparation can be achieved without the use of any additional solvents.

### 2.4. Three-Roll Mill

Samples with different types and amounts of nanofiller were dispersed on a three-roll mill (TRM) from the company Exakt (Exakt 80E Plus, Exact Advanced Technologies GmbH, Norderstedt, Germany). Prior to the dispersion routine, the CNTs and CNFs were pre-dispersed into the CR141 resin by a mechanical stirrer for 5 min. Subsequently, we started the TRM-process, which was sequenced into 4 different parts: starting from a gap size of 120 µm at the first gap and 40 µm at the second ca,p the gap sizes were reduced after each run (Table 1). In the last step, the mode of operation was changed from gap size controlled to force-controlled, with the following parameters: first gap 5 µm and at the second gap, nominally at 0 µm but with a constant line pressure of 5N/mm. The curves of the resulting shear forces over time were recorded by the TRM Exakt software Datalog Plus for all samples. The semi-dispersed sample was produced by application of steps one and two only. The dispersion process of one sample took about three to four hours.

### 2.5. Ultrasonication

**Figure 1:**
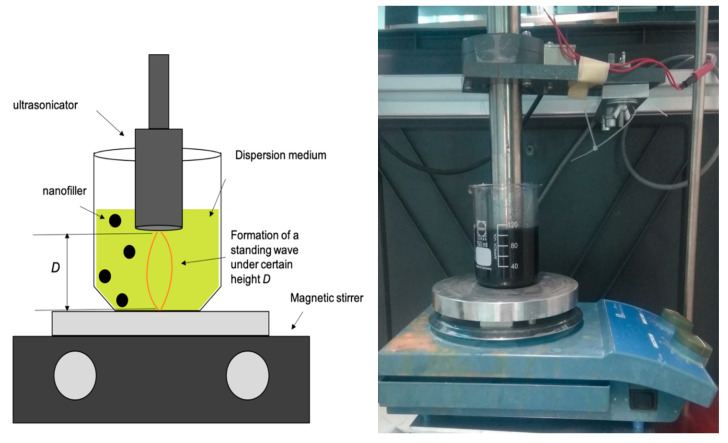
Schematic experimental setup of the ultrasonic dispersion experiments.

The second applied dispersion method was ultrasonication. Due to a mixing ratio (in wt%) of 10 parts resin to 9 parts hardener, it is possible to disperse the nanofillers by sonication in CH141, which provides a way lower viscosity compared to CR141. We used a self-constructed ultrasonication setup together with a magnetic stirrer. The ultrasonic sonotrode induced vibrations with a frequency of about 18.9 kHz with about 10 W power for 6 × 5 min (5 min pulse followed by 5 min pause resulting in total 30 min ultrasonication) to avoid heating (Figure 1). Overwhelming heating of the matrix could be avoided by the pulse-pause scheme and was tested by a measurement with a thermo couple: during the pulse period of 5 min, the temperature increased from 24.8 °C to 41.3 °C, followed by a cooling down to 26.7 °C during the pause period of 5 min. The magnetic stirring helped to avoid the formation of any hot spots during sonication. The semi-dispersed sample was produced with a sonication time of only 3 × 5 min without magnetic stirring.

The basic relation between the speed of sound *c_s_*, frequency *f,* and wavelength *λ* for a monochromatic wavelength is given by:

(1) $$c_{s}=\lambda \cdot f$$

In a fluid, the speed of sound can be calculated if the material properties are known, by:

(2) $$c_{s}=\sqrt{\frac{K}{\rho }},$$

where *K* represents the bulk modulus and *ρ* the density of the fluid. Under certain conditions in a closed system with the length *D*, the sound waves can form standing waves:

(3) $$D=\frac{\lambda \cdot n}{2}$$

where *n* ∈ ℕ is a natural number. Thus, depending on the density of the used fluid, certain immersion depths of the sonicator could cause the formation of standing waves and thus hinder a proper dispersion of the nanotubes. Therefore, the sonicator has to be positioned away from such nodes. This could be done after the measurement of the speed of sound and calculation of *D*.

The speed of sound was determined in an ultrasonic reflection measurement. The setup consists of a sample chamber, which is completely filled with the testing fluid, a sensor carrier, and, on top, a mounted ultrasonic sensor (OPBOX 2.0, Optel Ulrtrasonic Technology, Wroclaw, Poland). The schematic setup can be found in the Supplemental Information in Figure S18. After the complete filling of the sample chamber with the hardener, it is closed by the sensor carrier made of steel, with the ultrasonic sensor on top. The ultrasonic sensor is used both as emitter and receiver. By closing the sample chamber, the excess of hardener can flow off. The sensor emits an ultrasonic pulse that travels through the sensor carrier plate. Most of the pulse is reflected at the interface between sensor carrier and liquid and returns to the receiver. Only a small pulse runs through the fluid, is reflected at the bottom of the sample chamber, returns through the chamber and the carrier, and is also detected at the receiver. The transit time difference of the two signals is dependent on the speed of sound in the liquid and the additional travel path of the sound in the liquid, i.e., two times the height of the sample chamber.

### 2.6. Microscopy

Microscopy images were taken with a Keyence VHX 5000 light microscope at different magnifications (500- and 1000-fold). The size of agglomerates was measured, and the dispersion quality evaluated. Therefore, a processing routine with the open-source image processing package Fiji, which is based on imageJ (developer Wayne Rasband, NIH, Bethesda, USA), was developed. The grey values of the 8-bit microscopy images were set to a certain threshold, and after that, binary images were created. In this way, we were able to generate a picture of “inclusions” which resemble the carbon nanotube agglomerations in the dispersion. We then used a built-in particle analysis function, which fits the outlines of the agglomerates and evaluates the area (in the calibrated unit) of the particles, the Feret diameter, which is the longest distance between any two points in the boundary [27] and was used in other studies to analyze the sizes of nanoparticles [28,29,39] and the circularity (Fiji-formula: $\frac{4\pi *area}{perimeter^{2}}$), which assesses the shape of the agglomerates: 1.0 would be a perfect circle, whilst a value approaching 0 indicates an elongated shape. The evaluation range for the area was 0.1 to 700 µm^2^, for the Feret diameter, it was 0.5 to 100 µm, both selected from the microscopy images. The dispersed material used for microscopical analysis was pulled out from the middle section of the sample without additional stirring. All samples were tested within 48 h after the dispersion process.

### 2.7. Rheology

Rheological tests were performed on an MCR 300 rheometer (Anton Paar Austria GmbH, Graz, Austria) with a plate-plate system with a plate diameter of 25 mm and a working distance of 0.5 mm at 25 °C. The rheometer gap was filled in the same procedure with a slight overfill without trimming. Measurements were carried out in rotational shear mode and in oscillatory shear mode. Amplitude sweeps were carried out at 10 rad/s and strain values from 0.1 to 100% to determine the linear viscoelastic regime (LVE) of the samples. Frequency sweep measurements were performed between 1 and 100 rad/s in the sample LVE, which was between a strain value of 0.2% for US dispersion samples and 0.5% for TRM samples. Rotational tests were performed at shear rates from 0.01 to 100 1/s and 0.001 to 10 1/s. All samples were tested within the same time range of 24 h after dispersion via TRM or US to avoid possible sedimentation prior to the rheological measurements.

## 3. Results

### 3.1. Oxidation State of the CNT by XPS

The oxidation state of the CNT after H_2_O_2_ treatment at room temperature (RT) for a week was measured by XPS (K-Alpha XPS system, Thermo Fisher Scientific, UK). Samples produced with the new method were compared to samples produced at elevated temperature (ET). An untreated sample of CNT was measured as control (C). XPS spectra of these CNT are presented in Figure S8. The XPS results can be found in the Supporting Information, in Table S1. The degree of oxidation was 0.1 at% for the control, 2.7 at% for ET sample, and 2.4 at% for the RT sample. The measurements show that the RT method yields a degree of oxidation comparable to the one obtained by the ET method.

### 3.2. Determination of Hardeners’ Speed of Sound and Optimal Sonicator Height

**Figure 2:**
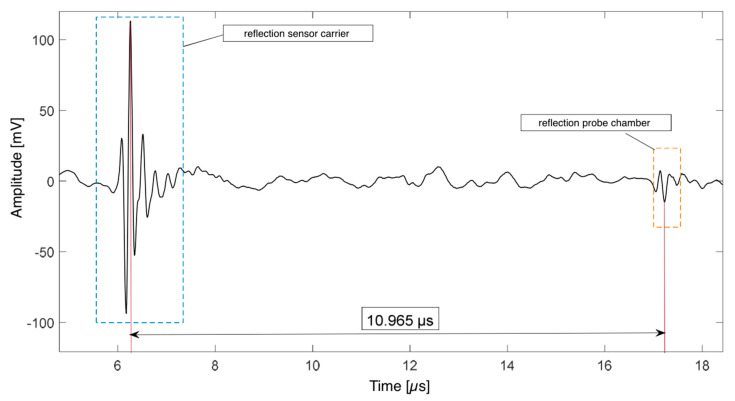
Reflected ultrasonic pulse signals at the boundary sensor carrier/probe and probe/probe carrier.

The density of the hardener is *ρ* = 1.195 g/mL and was taken from the data sheet of the supplier. The speed of sound could be determined in the ultrasonic reflection measurements. The travelled path of the ultrasonic pulse was twice the chamber height of (8.29 ± 0.1) mm. Thus, the total covered path is $s_{path}=\;2\cdot s_{chamber}\;=16.58\;\pm 0.2\;\mathrm{mm}$ The frequency of pulses was 100/s and the measurement time was 15 s, i.e., the resulting signal is an average of 1500 single measurements. The sound runtime was $t_{path}=10.965\;\mu s$ (Figure 2).

The speed of sound was calculated by

(4) $$c_{s}=\frac{s_{path}}{t_{path}}$$

and was *c_s_* = (1512 ± 2) m/s. With this value, the wavelength (Equation (1)) and subsequently, the immersing depth (Equation (3)), which would induce nodes, were calculated (Table 2).

We chose a height of 30 mm, which is far away from 40 mm, for a first test. A value below 40 was chosen because of the amount of hardener in this test series and therefore the height of the beaker was low. To find the best setup, we varied D from 20 to 30 mm in 2.5 mm steps. From each dispersion, microscopic images were taken. Thus, we were able to select an immersion depth of 27.5 mm for optimal results (example images can be found in the Supplemental Figure S1), because between 25 and 30 mm, no big difference was observed.

### 3.3. Microscopic Characterization of TRM and US Dispersions

**Figure 3:**
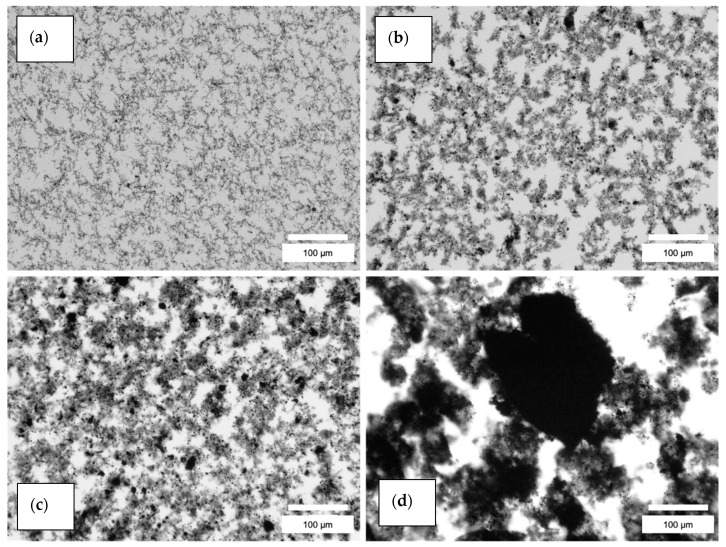
Comparison of different nanofillers dispersed in hardener (ultrasonication—US): (**a**) 0.5 wt% carbon nanofiber (CNF); (**b**) 0.5 wt% CNToxi; (**c**) 0.5 wt% carbon nanotube (CNT); (**d**) 0.5 wt% CNT semi-dispersed.

**Figure 4:**
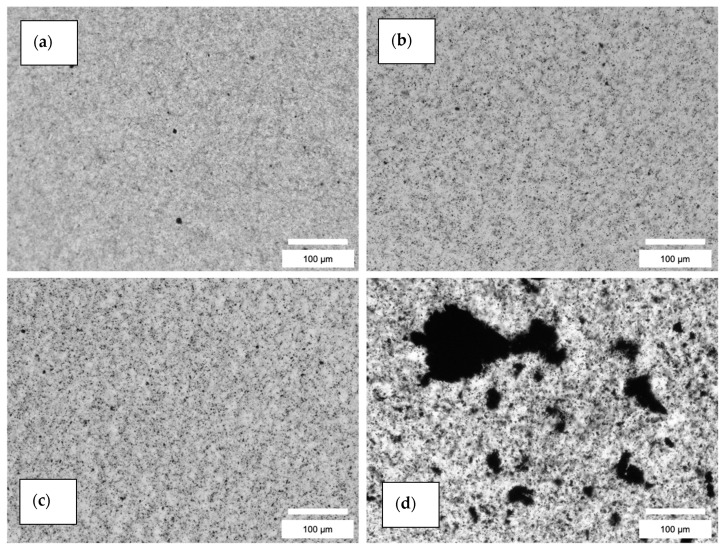
Comparison of different nanofiller dispersed in resin (TRM): (**a**) 0.5 wt% CNF; (**b**) 0.5 wt% CNToxi; (**c**) 0.5 wt% CNT; (**d**) 0.5 wt% CNT semi-dispersed.

**Figure 5:**
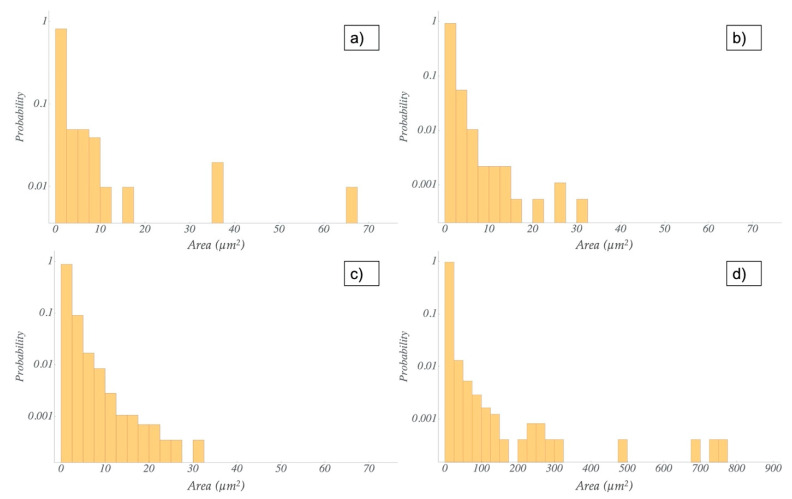
Comparison of the distribution of agglomerate area size for the TRM dispersion for the 0.5 wt% filler grade and different fillers: (**a**) CNF; (**b**) CNToxi; (**c**) CNT; (**d**) CNT semi.

**Figure 6:**
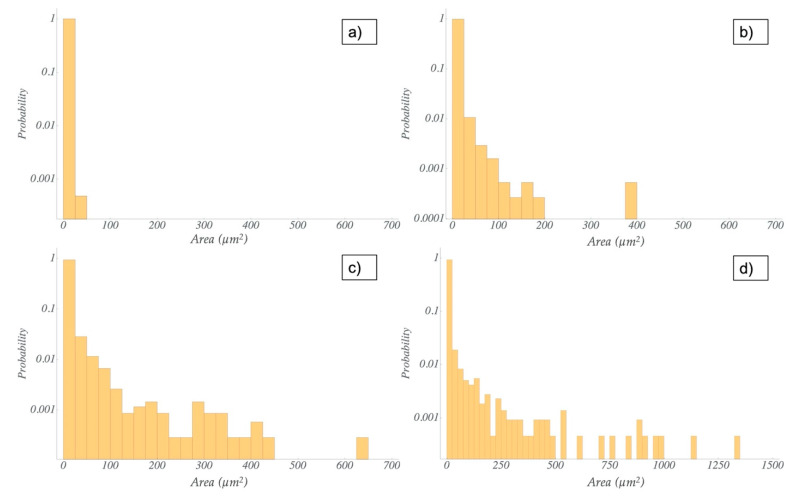
Comparison of the distribution of the agglomerate area size for the US dispersion for the 0.5 wt% filler grade and different filler: (**a**) CNF; (**b**) CNToxi; (**c**) CNT; (**d**) CNT semi.

The agglomerate distributions resulting from both dispersion methods were analyzed by the fitting of area sizes and the Feret diameter of the particles. The microscopic images showed a better dispersion of the nanofillers in the resin via the TRM compared to the dispersion in the hardener via US (Figure 3, Figure 4 and Figures S11–S16). For the latter, some larger agglomerates were also found. Nevertheless, the histograms for both methods (Figure 5 and Figure 6) reveal that most of the agglomerates are in an area range below 100 µm^2^ for the lowest filler grade of 0.5 wt%. With increasing filler grades, in both systems, the agglomerate size distributions were broadening (Figures S2–S7). Both semi-dispersed samples showed larger area distributions and bigger maximum agglomerate sizes.

**Figure 7:**
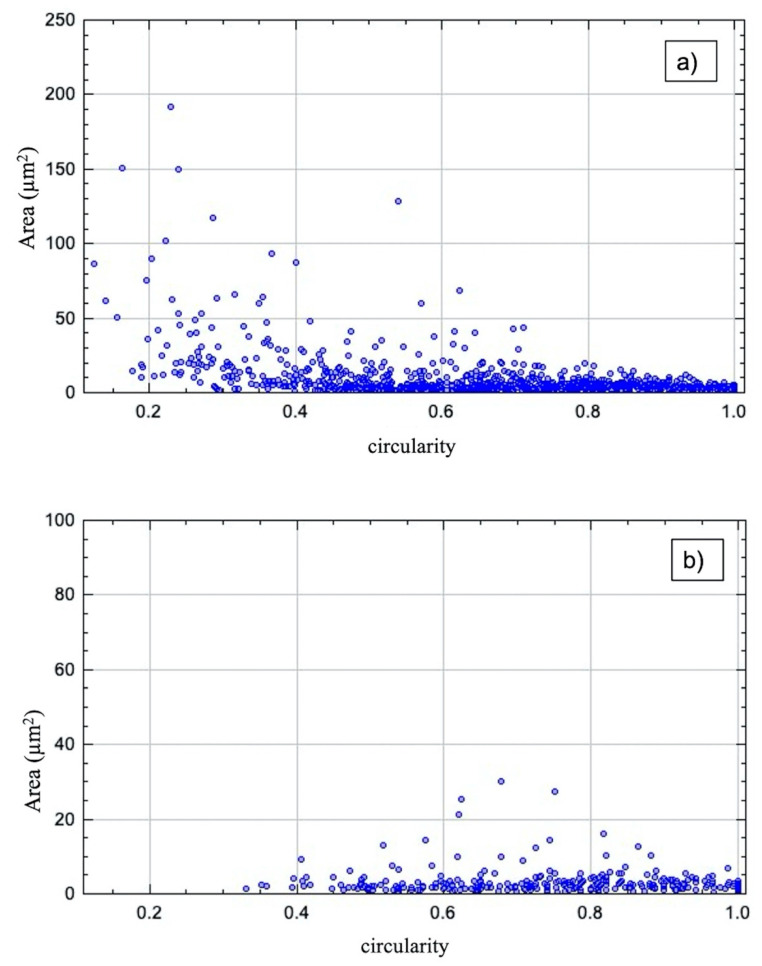
Comparison of (**a**) US and (**b**) TRM dispersion of 0.5 wt% CNToxi of the circularity in reliance to the area in µm^2^. Area errors can be estimated through the grey value evaluation of the microscopic images in imageJ with about 1% of the values given. The two plots feature different scaling of the *y*-axis.

For all area values, we evaluated the corresponding circularity values. Values of 1 represent a perfect circle, values towards 0 refer to more elongated agglomerates. For both dispersion methods, most of the agglomerates showed a circularity in the range of 0.5–1. Especially in the US samples, larger agglomerates showed a smaller circularity value, i.e., elongated shape. Figure 7 shows, as an example, the comparison between the 0.5 wt% CNToxi US and TRM dispersion sample. Figure 7 also shows no agglomerates bigger than 40 μm^2^ area for the TRM sample while the US sample shows several bigger agglomerates in the range from 40–200 μm^2^ area.

We evaluated the Feret diameter, defined as the longest distance between any two points in the agglomerate. All values are shown in Table 3. For quantitative comparison of the size distributions, we defined two parameters, read from the histograms. The biggest agglomerate size from the continuous size distribution was defined as the cut-off, i.e., nearly all of the agglomerates are smaller than this value. The other one is the biggest agglomerate found in the evaluation range. The cut-off value increases for higher filler grades in all systems. Comparing the same amount of the same type of nanofiller, the TRM samples showed smaller cut-off and smaller maximum values than the US samples, with the only exception of the 0.5 and 1.0 wt% CNF samples. For these, both dispersion methods resulted in similar cut-off and maximum values of the Feret diameter. Not surprisingly, the semi-dispersed systems showed the highest values for the cut-off and the maximum Feret diameter.

### 3.4. Rheological Analysis

**Figure 8:**
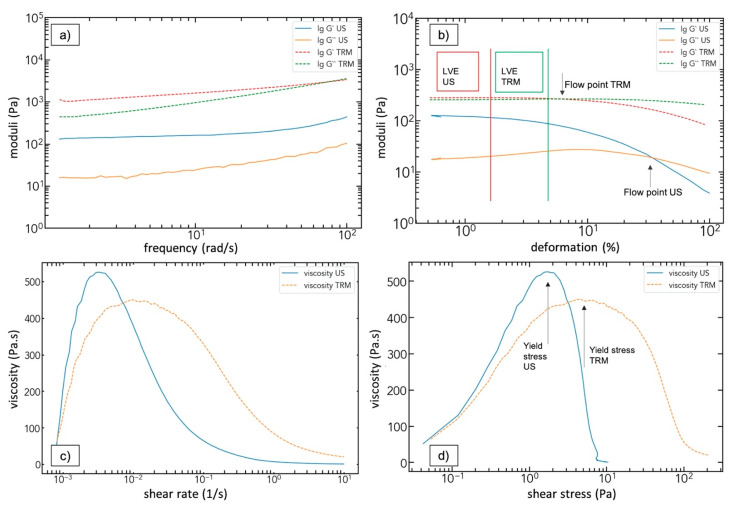
Overview of the different applied rheological tests and the resulting plots shown for the CNT 0.5 wt% US sample: (**a**) frequency sweep and the comparison of US and TRM dispersion; (**b**) amplitude sweep indicating the linear viscoelastic regime (LVE) and the flow point; (**c**) viscosity measurement showing the shear thinning behavior; (**d**) shear stress and the corresponding yield stress.

Figure 8 shows an overview of the curves that were obtained from the rheological tests and were the basis for further evaluation, such as the long-term stability or the viscoelastic behavior of our samples.

Oscillation tests revealed the linear viscoelastic regime (LVE) of the dispersion samples by applying an amplitude sweep (10 rad/s and strain values from 0.1 to 100%, Figure 8b and Figure S10). This regime is defined by a constant trend of the storage modulus G’ and the loss modulus G’’. The first describes the elastic portion of the sample, internal structures can be deformed without destroying the network itself. G’’ describes the viscous portion, which means the fluid behavior of the sample, which can be seen as energy lost through friction. A drop of one of the two curves marks the end of the LVE; the corresponding deformation value is defined as the yield point [40]. Beyond that point, irreversible structural changes occur inside the sample, whereas inside the LVE, those are all reversible. The second point found in the amplitude sweep is the so-called flow point, where the crossover of G’ and G’’ marks a turning point in the viscoelastic properties: G’ > G’’ describes a viscoelastic solid character in the LVE, G’ < G’’ represents a viscoelastic fluid sample [33,40]. All values found for the yield and flow points are shown in Table 4.

For most US samples, the yield point was about γ = 1%. In comparison, the corresponding TRM samples showed higher values for all filler grades and filler types, e.g., while CNT-US 0.5 wt% features γ = 1.27%, CNT-TRM 0.5 wt% features γ = 5.7%. For the TRM samples, a decrease of the yield point with increasing amount of fillers could be observed for all filler types. For the US samples, no clear trend was found, except the CNToxi series. The turning point of the viscoelastic properties, defined by the flow point, was found at higher deformation values for all US samples compared to the TRM samples. The CNF-TRM samples constitute an exception, where no viscoelastic turning point was found.

**Figure 9:**
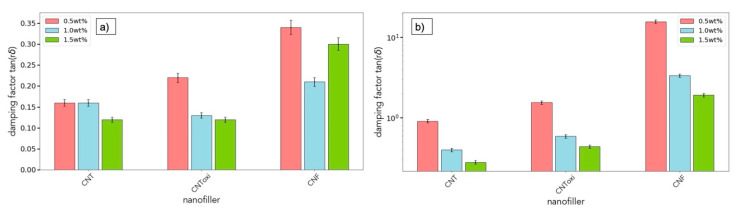
Damping factor shown for both dispersion methods and all filler and filler grades. (**a**) US dispersion (**b**) TRM dispersion. Note: the TRM dispersion plot is evaluated with a logarithmic *y*-axis.

An additional feature evaluated via the amplitude sweep is the so-called damping factor tan(δ). It is defined as the phase difference δ between G’’ and G’ and can be calculated by tan(δ) = $\frac{G^{\prime \prime }}{G^{\prime }}$. This value has to be calculated inside the LVE regime of a specimen and allows to estimate the possibility of sedimentation inside the sample; values < 1 counteract this effect and avoid structural damages [41,42]. Sedimentation is controlled by various effects, dominated by viscosity, but also, other influences like agglomerate size, interlinks, or buoyancy can play a role. The values of G’ and G’’ were taken at a specific deformation value of γ = 0.2%, because in the LVE, those values are constant anyhow. Results can be found in Figure 9.

In all US dispersion specimens, the damping factor was clearly < 1, it never exceeded a value of 0.35. For the TRM samples, for all fillers, tan(δ) decreased with an increasing filler amount. With respect to the values of the 0.5 wt% CNT and 0.5wt% CNToxi, which are close to 1, higher amounts of these nanofiller types display values smaller than 1. The CNF show much higher values of tan(δ), i.e., larger than 2. Sedimentation can also occur over a longer time period. Due to this fact, we additionally analyzed the damping factor through a frequency sweep for a lower frequency value of 2 rad/s and γ = 0.2%. These values delivered the same outcome and were also numerically very close to what was found through the amplitude sweep (Figure 9) and can be found in the Supplemental Information in Table S3.

An oscillatory frequency sweep (Figure S9) at a constant amplitude depending on the LVE range limits of the samples (γ = 0.5% for TRM, γ = 0.2% for US) was applied. This test was mainly used to gain insights in the long-term behavior of the dispersions, regarding sedimentation and settling. In a perfectly stable dispersion, the curves of G’ and G’’ show a slight slope and a parallel progress over the whole measurement area; the ratio of G’ and G’’ are in a range of 10:1 to 100:1 [40]. Table 5 shows these parameters. The slopes ∆G’/∆f and ∆G’’/∆f were evaluated over the whole measurement area ranging from 1 rad/s to 100 rad/s. Values for G’ were evaluated in the low-frequency area at a value of 2 rad/s; the corresponding table can be found in the Supplemental (Table S2). Since all curves showed a highly parallel progress over the measurement areas, G’:G’’ was also analyzed at 2 rad/s. All ratios are below the ideal region, but still, the requirement G’ > G’’ for a stable dispersion is given for all US samples. All CNF-TRM and the 0.5 wt% CNToxi-TRM sample showed a viscoelastic liquid character with G’ < G’’. The remaining TRM-samples were characterized as viscoelastic solid. All TRM samples featured lower storage to loss modulus ratios and steeper slopes of these parameter curves compared to the corresponding US samples.

**Figure 10:**
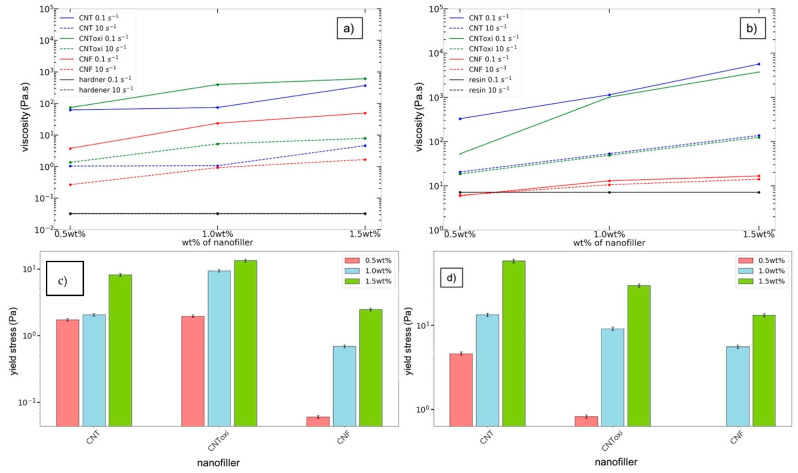
Viscosity values for all nanofillers and filler grades at different shear rate values showing the shear-thinning behavior. (**a**) US dispersions; (**b**) TRM dispersion. Yield stress evaluated through rotational shear rate-controlled tests (**c**) US dispersion; (**d**) TRM dispersion. Errors for the viscosity values and the yield stress are estimated to be 5% due to the evaluation process.

Rotational tests were performed in order to determine the viscosity values and to determine the shear-thinning character of the samples; both can be used to characterize the dispersion quality [36,43]. The resin and hardener showed Newtonian behavior and the viscosity curves for both can be found in the Supplemental Information in Figure S21. The CNToxi-US samples showed for all filler grades the highest viscosity values at a shear rate of 0.1 s^−1^; the lowest were found for the CNF samples. All CNT-TRM samples showed the highest viscosity values at 0.1 s^−1^ compared to the other filler types; the lowest values were again found for the CNF samples. With higher filler amounts, the viscosity values increased for all nanofiller types for the US dispersions, and also the drop of the viscosity values between the shear rates of 0.1 and 10 s^−1^ were more pronounced with higher filler amounts (Figure 10a,b). The CNF-TRM samples were an exception to this: the 0.5 wt% sample did not show any shear-thinning behavior, while higher filler amounts only exhibited slight changes in viscosity for increasing shear rates. CNT-TRM and CNToxi-TRM followed the shear-thinning behavior also observed in the US samples.

Further, the yield stress was determined (Figure 10c,d) and provides another characterization tool for the dispersions. The yield stress is nevertheless not a material constant since it mainly depends on the testing method and the measurement setup. It should just be seen as an additional estimation and only as a comparison between the samples and the dispersion methods concerning the stability of the system. In the US dispersion series, we found increasing yield stress values for increasing filler amount for all systems, in which the CNToxi group showed the highest values compared to the normal CNT and CNF samples. The same is true for the TRM dispersions; the only sample for which we found no yield stress was the 0.5 wt% CNF sample. This sample showed constant viscosity rates over the whole testing range.

The samples of each matrix system were prepared once. Nevertheless, the data is still representative, the rheological tests deliver integral parameter, and the microscopic size fitting features a high number of agglomerates and is thus statistically relevant. The errors for all parameters were estimated from the evaluation process and Gaussian error propagation calculations. The error in the histograms can be estimated through the grey value evaluation of the microscopic images in imageJ with about 1% of the values given.

## 4. Discussion

In the course of this study, we were able to disperse different nanofillers directly in hardener via an ultrasonic dispersion procedure. Other studies applied this method only in combination with solvents, where the nanofillers were dispersed in, e.g., acetone prior to the dispersion in the matrix. Consequently, the solvent had to evaporate completely over time or there were additional steps, e.g., heating of the sample must be applied [21,23]. Such an approach has two main disadvantages: the work with a high amount of acetone, xylene, or comparable constitutes a health risk for the people working with it, and further, it is not known if the whole amount of used solvents can evaporate and whether those have an unwanted effect on the nanofillers or the dispersion matrix itself. Nevertheless, with a sufficient amount of a low-viscosity matrix component (in our case, the hardener), we could show that direct dispersion is possible. Dispersion of nanofillers in hardener by TRM or dispersion of nanofillers in resin by US were not applied due to several reasons: due to high viscosity of the resin, a successful US dispersion would only be possible with additional solvents, whilst a dispersion in hardener via TRM would hardly be possible due to the low viscosity of the system. Additionally, later mixing of resin and hardener and curing will follow the same procedure with a weight ratio of 10:9. For this reason, the results of both dispersion methods can be compared on the level of first dispersion, i.e., resin or hardener alone, with the nanofiller.

Ultrasonic dispersion process could lead to a shortening of the nanofillers in length, and thus a reduced aspect ratio, that would influence the dispersibility. Several studies investigated the shortening of CNT through ultrasonic treatment [18,19]. A shortening in length by 36% was discovered after ultrasonication for 60 min and a power of 200 W [18], reduction in length by even 82% after 7 h at 100 W [19]. Length reductions due to sonication at a short dispersion time were observed for 40 W [20]. Nevertheless, in our study, a power of about 10 W was applied for 30 min during ultrasonication, which makes it likely that no considerable shortening or damage of the CNT occurred. A systematic study including XPS measurements will clarify this. For checking on a possible length reduction in our experiments, we analyzed samples in the following way: resin and hardener with dispersed CNT, CNToxi, and CNF were diluted with acetone, and the nanofillers were filtered and dried. CNT samples were investigated by transmission electron microscopy and CNF samples by scanning electron microscopy. The resulting micrographs can be found in the Supplemental Information (Figures S19 and S20). Those images show no qualitative difference between neat, TRM, and US treated nanofillers. Preliminary tests showed a breakdown of the standing wave by increased sonication power, caused by a change in mean system density and thus speed of sound due to an increase in cavitation. This might increase dispersion quality by avoiding the formation of a standing wave but would at the same time enhance damage of the nanofillers [18]. Therefore, the comparable low power of about 10 W was applied. Thus, the height of the sonicator had to be adjusted and magnetic stirring was applied in addition to facilitate dispersion and avoid compactification of the nanofillers by the standing wave. Thus, we conclude that no considerable reduction in length of the nanofillers occurred during our experiments.

Comparing the different dispersion methods, the ultrasonic dispersions showed higher values of agglomerate sizes and Feret diameters in the CNT and CNToxi samples than the TRM-produced dispersions. The CNF samples showed almost the same values for the two lower filler amounts. For the CNToxi-US 0.5 wt% sample, most of the agglomerates had Feret diameters below 20 µm, while the CNToxi-TRM 0.5 wt% sample showed values below 10 µm. These values may seem high but are found with an extremely low probability (Figure 6), while most of the agglomerates are in a size range of below 5 µm. Additionally, the fitting routine could not always discern closely associated agglomerates in US dispersions with higher filler amounts due to resolution restrictions and fitted them as one single agglomerate. This also leads to higher values for the Feret diameter and the area. The probability of the occurrence of large agglomerates is nevertheless extremely low, i.e., most of the particles are well dispersed, especially for the lowest filler amount of 0.5 wt%. The circularity values of agglomerates in the US samples were lowest for larger agglomerates, which could be a hint at a loose association of the nanotubes, rather than the formation of real agglomerates. In a previous study, MWCNT were partly dispersed in an ultrasonic bath only, resulting in dispersions with agglomerate sizes up to 100 µm. Nevertheless, reinforced composites produced from this matrix showed mechanical properties comparable to samples produced with dispersions from the TRM, i.e., also a certain amount of agglomerates larger than 20 µm had no negative influence on tensile strength, Youngs modulus, and flexural strength. [12]. Wu et al. dispersed MWCNT in epoxy resin by mechanically stirring the dispersion for 4 h followed by 1 h sonication. Microscopy images of these dispersions showed agglomerates with estimated diameters of about 25 µm; the mechanical properties of the reinforced materials where nevertheless improved [44]. Additionally, most of the literature applying ultrasonic dispersion with additional solvents do not report on the dispersion quality. The agglomerate sizes are hardly analyzed and can only be estimated via microscopy images [22] or SEM pictures [21]. In two studies, in which nanofillers are dispersed directly in an epoxy-hardener matrix, quality control is only accomplished by SEM images [25,26]. In the present study, we were able to evaluate the size distributions of all dispersion samples and thus, quantitatively analyze the effectiveness of our dispersion method.

For enhancement of the dispersion quality, oxidation of CNT was chosen. This process induces hydroxyl, carbonyl, and carboxyl groups on the surface. Functionalization of the CNT could improve the linking of the nanotubes to the matrix noticeably [12,36]. In our work an oxidation degree of 2.4% for the RT functionalization process was reached. This is in the desired optimal range of a maximum of 5% to maintain all the CNT properties and enhance the interaction with the matrix through the provided functional groups [12]. For both dispersion methods, the oxidized CNT showed narrower agglomerate size distributions as compared to the conventional CNT for the same filler amount and represent thus the better attachment to hardener and resin, respectively.

Besides microscopy, rheology is a very powerful method for probing the dispersion quality. Amplitude sweeps were used to determine the LVE range and the flow point (Table 4), the limit of the LVE can be seen as the maximum of deformation which can be tolerated by the system before the internal structures break down [40]. The TRM samples showed higher LVE areas and thus are more stable to mechanical deformation. However, the CNF TRM-dispersed samples showed a viscoelastic liquid character over the whole measurement, with no clear yield point and no flow point. The US-dispersed ones were, like all other samples, viscoelastic solid to a certain extent with a defined yield and flow point. We can thus link the LVE with the dispersion quality via microscopy images. Well dispersed CNT samples will show small agglomerates in microscopy as well as viscoelastic solid character with defined yield and flow point. For CNF samples the picture is more complex: The TRM-CNF and US-CNF samples show good dispersions in the microscopy, although TRM-CNF show smaller agglomerates. Rheology shows the difference in entanglement with respect to CNT nanofillers: TRM-CNF samples display viscoelastic liquid character, i.e., well separated CNFs, while the US-CNF samples revealed a viscoelastic solid character. This can be explained by a higher degree of entanglement, due to the dispersion method itself: calendaring through the TRM achieves nevertheless a better separation of the CNF agglomerates and more separate CNFs throughout the matrix.

The long-term stability and the quality of the dispersions can further be analyzed via frequency sweeps. Particles in dispersion are exposed permanently to gravity. To hinder or decelerate sedimentation, counterforces must be built up inside the dispersions. Other studies found that the formation of an internal percolation network requires that G’ > G’’, i.e., G’:G’’ > 1 for frequency sweeps, otherwise viscoelastic liquid responses and a relaxation behavior is still dominant [33,40]. We found for all US samples, that G’:G’’ > 1, while some TRM-samples showed different behavior. For the 0.5 wt% TRM-CNToxi sample, we found a well-dispersed state with more uniformly distributed CNT under the microscope, but since G’:G’’ < 1, this is indicating a higher probability of sedimentation. Higher filler amounts of the TRM-CNToxi samples showed slightly larger agglomerates but also a moduli ratio > 1, indicating a network formation. Additionally, all CNF-TRM samples showed a rather uniform distribution under the microscope, but also G’:G’’ < 1. Thus, we can say that a uniformly distributed state with hardly any agglomerate classifies such samples as well-dispersed under the microscope, but from a rheological point of view, those samples show higher probabilities of sedimentation. Samples with uniformly distributed nanofillers but also a higher amount of small agglomerates can form a percolation network and hinder sedimentation.

Pötschke et al. stated that G’ and G’’ can be almost frequency-independent at low frequencies, where this effect is more pronounced for the storage modulus, and linked this with the formation of internal structures [45]. In this study, the curves of G’ and G’’ were almost frequency-independent at low frequencies in all US samples, while the TRM samples showed steady slopes over the whole frequency range. We conclude that microscopically best-dispersed samples (Figure 4), which show a high amount of uniformly distributed nanofiller i.e., the TRM samples, are less likely to form internal networks compared to the US samples, and thus show an increased probability of sedimentation. Opposite to that, the US samples, which revealed larger agglomerates, sometimes “cloudy” structures, and loose entanglements (Figure 3), rheologically represent dispersions with a strong percolation network.

Wang et al. found for poly(butylene succinate)/MWCNT dispersions that the slopes of G’ and G’’ decrease with increasing filler amount and connected these findings with increasing polymer-nanotube or nanotube-nanotube interactions and the formation of a network-like structure [34]. In contrast to that, stable dispersion was also characterized by a parallel course of the G’ and G’’ curves with a slightly increasing slope over the whole frequency range [40]. We found an increase of the slopes with increasing filler amount for both methods. The TRM samples showed steeper slopes compared to the US samples. The values found for all 0.5 wt% US samples are comparable to those found by Wang et al. Additionally, from this, we can conclude that a slightly better long-term stability can be assumed in the US samples compared to the TRM samples.

It was stated before that rheological parameter, e.g., G’, can directly be used to evaluate the dispersion quality [46]. Fan et al. found that higher values for G’ in dispersion with the same filler amount indicate a better dispersed system due to an interconnecting MWCNT network and a higher separation of the MWCNT. Further, G’ is characterized to be sensitive to nanofiller interconnections [43]. In the US batch, the CNToxi samples showed for each filler grade the highest G’ values (bearing in mind, that for the 0.5 wt%, the differences between CNToxi and neat CNT were minimal), while the CNF sample values were lowest (G’ values found in Supplemental Table S2). In the TRM sample batch, the CNT samples revealed the highest values for each filler amount, again, all CNF samples had the lowest values for G’. This would indicate more pronounced nanofiller interconnections in the US-CNT samples, while for the TRM dispersions, the neat CNT samples showed rheologically the most interconnections. With increasing filler amounts, the storage modulus increased for both US and TRM samples. It was stated in a study by Song et al. that this is due to the high aspect ratio and surface area of the nanofillers, which raises the storage modulus leading to the formation of a percolation structure [35]. It was further reported that even for low filler amounts, small sized particles easily form strong particle-polymer interactions and thus a physical network structure due to an increased interfacial area between particle and polymer [47,48]. Kim et al. ultrasonically dispersed MWCNT and functionalized MWCNT in epoxy resin by the use of acetone as additional solvent and analyzed the viscosity behavior of the dispersions in rotational tests [36]. They found a strong shear-thinning behavior of all samples, which was even more pronounced in the functionalized samples. Kim et al. explained these findings by an interfacial bonding of the MWCNT to the epoxy resin, which is more effective for surface modified CNT. In this study, US-CNToxi samples showed the strongest shear-thinning behavior, while for TRM, the neat CNT samples were revealed to have the highest shear-thinning behavior (see Figure 10). The only sample series that showed almost no shear-thinning behavior was the CNF-TRM sample. Additionally, we applied a 3ITT test (rotational test: 100 s at a shear rate of 0.1 s^−1^, followed by 80 s at 100 s^−1^, and a recovery phase of 130 s at 0.1 s^−1^) on the 0.5 wt% CNToxi US and TRM sample. Both samples revealed thixotropic behavior: shear-thinning, but also the recovery process of the inner structures were more pronounced in the US sample. The corresponding plot can be found in the Supplemental Information in Figure S17. Fan et al. stated that the viscosity η is more sensitive to nanofiller separation, meaning higher flow resistance is given when the nanofillers are more separated [43]. Again, we found highest values for each filler amount in the CNToxi samples and lowest for the CNF samples amongst the US samples. For the TRM sample batch, the CNT samples revealed the highest values and the CNF samples the lowest. Combining all these findings, we conclude that an interfacial bonding of the nanofillers to the matrix can be assumed for all samples except the TRM-CNF samples. This effect was more pronounced in all US samples and is a further indication for good dispersion quality. Considering the dispersion quality, microscopy images in combination with the frequency sweep and rotational test results of the parameter G’ and η show that the CNToxi samples delivered the best results in the US dispersion series. CNToxi samples showed an enhanced binding to the hardener and the best dispersibility compared to the untreated CNT and CNF nanofiller. The microscopical analysis showed very good dispersions for all TRM samples, which could be expected from this well-established method. The rheological results with respect to G’ and η show as well internal percolation networks for the CNT and CNToxi samples, indicating good dispersions. Interestingly, for the TRM sample series, the CNT samples revealed the best dispersion quality through the rheological tests, whilst the agglomerate size fitting of the microscopy images showed slightly better results for the CNToxi samples.

The rheological results indicate good dispersion quality of the US-dispersed samples in comparison to the established TRM dispersion method. These findings are further supported by the results of the amplitude sweep tests: Values of tan(δ) < 1 are indicating no sedimentation and thus a long-term stable dispersion [41]. We found tan(δ) values to be smaller than one for all samples for which a network formation is assumed, i.e., for all US samples, all TRM-CNT, 1.0wt% TRM-CNToxi, and 1.5wt% TRM-CNToxi. The tan(δ) value was bigger than one for those TRM-samples, for which liquid properties were found to be dominating, i.e., 0.5 wt% CNToxi and all CNF samples (values of tan(δ) can be found in Figure 9).

Combination of microscopy and rheology allowed an analysis of the nanofiller modified hardener system in the context of not only dispersion quality, but also processability and long-term stability. We could show that CNT and CNF do feature quite different dispersion characteristics. Well-dispersed CNT samples tend to form an internal network structure that allows for long-term stability of the dispersion. The CNF samples showed a good dispersion behavior, with hardly any agglomerates found in the microscopy images, but the rheological analysis showed that, maybe due to their aspect ratio and rigidity, no strong network was formed, especially for the TRM samples. Thus, it is assumed that no long-term stability is given and sedimentation over time is likely.

Direct US dispersion of nanofillers in the hardener systems without additional solvents was successfully achieved, and thus can be seen as an applicable method for a simple and straight-forward dispersion with the potential for industrial use. The dispersion of higher filler amounts can possibly be improved considering the increased agglomerate sizes. Comparison of the microscopy images of the semi-dispersed CNT sample and the 0.5 wt% CNT sample showed that only 15 min more sonication time deliver outstanding differences on the agglomerate size distributions. Thus, even higher sonication times will most probably still deliver better dispersions with lower agglomerate sizes for higher filler amounts. Further investigations will include analysis of possible shortening of the nanofillers by TEM and of changes of the oxidational state with XPS. Rheology and microscopy on the full epoxy system (hardener plus resin) will allow to state on the effect mixing has on the dispersion.

## 5. Conclusions

In this study, we were able to directly disperse various nanofiller types into a tetrahydromethylphthalic anhydride hardener via ultrasonication without the use of an additional solvent system. We analyzed the size distributions of the agglomerates and characterized rheological properties of the dispersions like estimation of long-term stability, sedimentation, and the viscoelastic behavior to evaluate the dispersion quality in depth and compared these results to TRM-produced samples. Especially, the 0.5 wt% oxidized CNT showed the best results of all US samples, revealing size distributions of most of the agglomerates in a comparable range to TRM-produced samples, a good binding to the hardener, the formation of a percolation network inside the dispersion, which points towards stability against sedimentation and thus a proper long-term stability. Higher filler amounts showed similar good results, but also the formation of more pronounced internal networks as well as somewhat larger agglomerates. CNF could also be dispersed successfully by the US method but dispersions of CNF in general are found to show a lower probability of forming a long-time stable system.

## Figures and Tables

**Table 1:** Overview of the used gap sizes for all three-roll mill processes.

TRM Gap	Step 1	Step 2	Step 3	Step 4	Step 4 Shear Force (N/mm)
Gap 1 size (µm) ± 1 µm	120	30	15	5	variable
Gap 2 size (µm) ± 1 µm	40	10	5	0	5

**Table 2:** Immersion depth D at which standing waves and thus nodes could occur with the given frequency in the CH141 hardener calculated with Equation (3).

n	D (mm)
1	40.0 ± 0.3
2	80.0 ± 0.5
3	120.0 ± 0.7

**Table 3:** Result of the Feret diameter evaluation: cut-off and maximum for different nanofillers and dispersion methods.

Filler Amount	CNT US	CNToxi US	CNF US	CNT TRM	CNToxi TRM	CNF TRM
	*Feret diameter Cut off* (µm) ± 2 μm					
0.5 wt%	44	21	9	9	8	6
1.0 wt%	50	32	14	20	11	11
1.5 wt%	49	36	19	22	10	20
0.5 wt% semi d.	80	/	/	69	/	/
	*Feret diameter Maximum* (µm) ± 2 μm					
0.5 wt%	48	47	9	13	9	11
1.0 wt%	71	59	14	24	18	13
1.5wt%	72	74	46	22	10	20
0.5 wt% semi d.	180	/	/	92	/	/

**Table 4:** Determination of the yield point and the flow point, representing the end of the LVE and the turnover of the viscoelastic behavior, respectively.

Filler Amount	CNT	CNToxi	CNF	CNT Semi	CNT	CNToxi	CNF	CNT Semi
Yield point (%)	Ultrasonication				Three-roll mill			
0.5 wt%	1.27 ± 0.06	1.40 ± 0.07	0.37 ± 0.02	1.08 ± 0.05	5.7 ± 0.3	3.8 ± 0.2	/	0.61 ± 0.03
1.0 wt%	0.29 ± 0.01	1.07 ± 0.05	1.43 ± 0.07	/	2.5 ± 0.2	2.1 ± 0.2	1.18 ± 0.06	/
1.5 wt%	0.93 ± 0.04	1.03 ± 0.05	0.20 ± 0.01	/	1.69 ± 0.08	1.60 ± 0.08	0.65 ± 0.03	/
Flow point (%)	Ultrasonication				Three-roll mill			
0.5 wt%	34 ± 4	23 ± 3	2.1 ± 0.3	24 ± 3	5.8 ± 0.6	/	/	8.9 ± 0.9
1.0 wt%	42 ± 5	27 ± 3	35 ± 4	/	27 ± 3	8.3 ± 0.9	/	/
1.5 wt%	37 ± 4	37 ± 4	1.6 ± 0.2	/	31 ± 4	11 ± 2	/	/

**Table 5:** Determination of the mean value and standard deviation of the G’:G’’ ratio and the slopes of G’ and G’’ over the whole measurement, which represents an estimation of the long-term stability of the dispersion samples.

Filler Amount	CNT		CNToxi		CNF		CNT		CNToxi		CNF	
G’:G”	$\frac{G^{\prime }}{G^{\prime \prime }}$ Ultrasonication at 2 rad/s						$\frac{G^{\prime }}{G^{\prime \prime }}$ Three-roll mill at 2 rad/s					
0.5 wt%	8.9 ± 0.5		6.9 ± 0.4		1.6 ± 0.1		2.1 ± 0.1		0.9 ± 0.1		0.01 ± 0.01	
1.0 wt%	7.1 ± 0.4		8.5 ± 0.5		5.9 ± 0.3		3.6 ± 0.2		1.9 ± 0.1		0.34 ± 0.02	
1.5 wt%	8.5 ± 0.5		8.8 ± 0.5		2.4 ± 0.2		4.7 ± 0.3		2.3 ± 0.2		0.67 ± 0.03	
	Moduli slopes Ultrasonication M						Moduli slopes Three-roll mill M					
slope	∆G’/∆f	∆G’’/∆f	∆G’/∆f	∆G’’/∆f	∆G’/∆f	∆G’’/∆f	∆G’/∆f	∆G’’/∆f	∆G’/∆f	∆G’’/∆f	∆G’/∆f	∆G’’/∆f
0.5 wt%	2.67 ± 0.05	0.85 ± 0.01	2.63 ± 0.03	0.87 ± 0.01	1.18 ± 0.03	0.58 ± 0.02	34 ± 1	24 ± 1	5.9 ± 0.2	15.4 ± 0.2	8.08 ± 0.04	0.8 ± 0.2
1.0 wt%	4.1 ± 0.3	1.05 ± 0.07	4.6 ± 0.2	1.54 ± 0.02	0.06 ± 0.01	0.61 ± 0.01	25 ± 2	24.6 ± 0.5	33 ± 1	33 ± 1	15.2 ± 0.2	5.42 ± 0.07
1.5 wt%	6.4 ± 0.4	2.24 ± 0.05	8.5 ± 0.1	2.21 ± 0.02	5.1 ± 0.1	1.32 ± 0.04	61 ± 3	41 ± 1	82 ± 3	57 ± 2	20.1 ± 0.3	8.3 ± 0.2

## Data Availability

Data is contained within the article or Supplementary Material.

## Supplementary Materials

The following are available online at https://www.mdpi.com/2073-4360/13/2/308/s1, Figure S1: Ultrasonication measurements of 0.5 wt% CNT at different sonicator heights to find optimal height of horn. Pictures show measurements at heights of 20 and 22.5 mm, Figure S2: Histogram agglomerate size distribution (in µm^2^) CNF 1.0 and 1.5 wt% US, Figure S3: Histogram agglomerate size distribution (in µm^2^) CNT 1.0 and 1.5 wt% US, Figure S4: Histogram agglomerate distribution (in µm^2^) CNToxi 1.0 and 1.5 wt% US, Figure S5: Histogram agglomerate size distribution (in µm^2^) CNF1.0 and 1.5 wt% TRM, Figure S6: Histogram agglomerate size distribution (in µm^2^) CNT 1.0 and 1.5 wt% TRM, Figure S7: Histogram agglomerate size distribution (in µm^2^) CNToxi 1.0 and 1.5 wt% TRM, Figure S8: XPS curves of CNT oxidized at RT (oxi 21), ET (CNT oxi 120°) and a reference sample, Figure S9: Example of frequency sweep plots for different filler grades of CNToxi for both, TRM and US: (a) storage modulus (b) loss modulus, Figure S10: example of amplitude sweep plots for different filler grades of CNToxi for both, TRM and US: (a) storage modulus (b) loss modulus, Figure S11: CNF 1.0 and 1.5 wt% US dispersion, Figure S12: CNToxi 1.0 and 1.5 wt% US dispersion, Figure S13: CNT 1.0 and 1.5 wt% US dispersion, Figure S14: CNF 1.0 and 1.5 wt% TRM dispersion, Figure S15: CNToxi 1.0 and 1.5 wt% TRM dispersion, Figure S16: CNT 1.0 and 1.5 wt% TRM dispersion, Figure S17: thixotropic behaviour evaluated for 0.5 wt% CNToxi US and TRM samples. Both samples showed the assumed thixotropic behaviour, which was more pronounced in the US sample, Figure S18: Setup of the felection measurement to determine the speed of sound in the used hardener matrix, Figure S19: TEM images of (a) CNToxi US, (b) CNT US, (c) CNT TRM, (d) neat CNT to check on possible length reduction and damages through the ultrasonic dispersion process, Figure S20: SEM images of (a) neat CNF and (b) CNF US to check on possible length reduction and damages through ultrasonication, Figure S21: viscosity plots for (a) hardener matrix and (b) resin matrix Table S1: XPS results of the CNT reference sample, The RT oxidized CNT and the 120° oxidized CNT, Table S2: Values for G’ at 2 rad/s for different filler grades of US and TRM dispersion, evaluated through frequency sweep tests, Table S3: Values for the damping factor at 2 rad/s for different filler grades of US and TRM dispersion.
