# Supplementary material for: Solvent-Free Ultrasonic Dispersion of Nanofillers in Epoxy Matrix
Source: Polymers (Basel). 2021 Jan 19;13(2):308. doi: 10.3390/polym13020308 (PMC7835899; doi:10.3390/polym13020308)
Supplement: Supplementary file 1 [file polymers-13-00308-s001.pdf]

polymers

MDPI

Supplementary

Solvent-Free Ultrasonic Dispersion of Nanofillers in Epoxy Matrix

Benjamin Zanghellini[1,*], Patrick Knaack[2], Sebastian Schörpf[2], Karl-Heinz Semlitsch[3], Helga Lichtenegger[1], Bernhard Praher[4], Maria Omastova[5] and Harald Rennhofer[1]

[1] Instiute of Physics and Materials Science, University of Natural Resources and Life Sciences Vienna, 1190 Vienna, Austria
[2] Instiute of Applied Synthetic Chemistry, Technical University of Vienna, 1190 Vienna, Austria
[3] Secar Technology GmbH, 8682 Mürzzuschlag, Austria
[4] Institute of Polymer Injection Moulding and Process Automation, Johannes Kepler University Linz, 4040 Linz, Austria
[5] Polymer Institute, Slovak Academy of Sciences, Dubravska cesta 9, 845 41 Bratislava, Slovakia
* Correspondence: benjamin.zanghellini@boku.ac.at

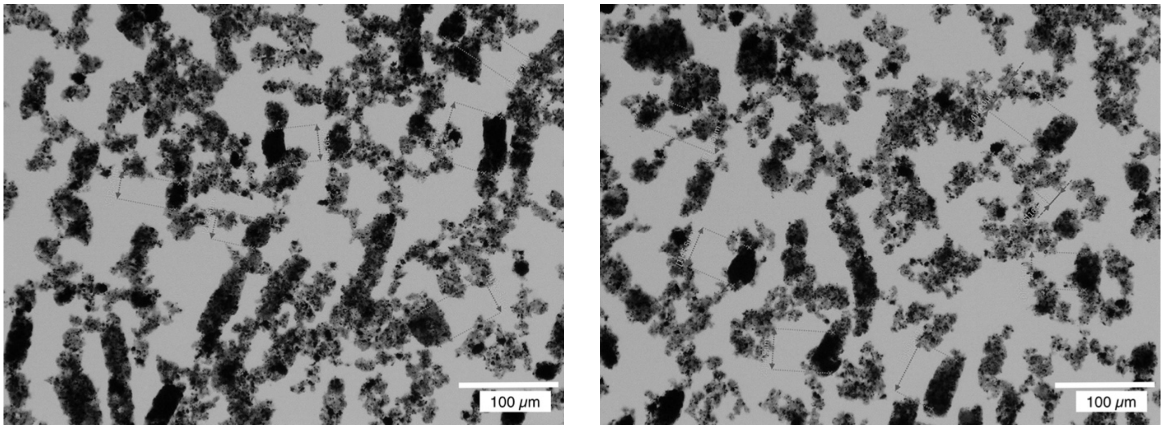

Figure S1. Ultrasonication measurements of 0.5 wt% CNT at different sonicator heights to find optimal height of horn. Pictures show measurements at heights of 20 and 22.5 mm.

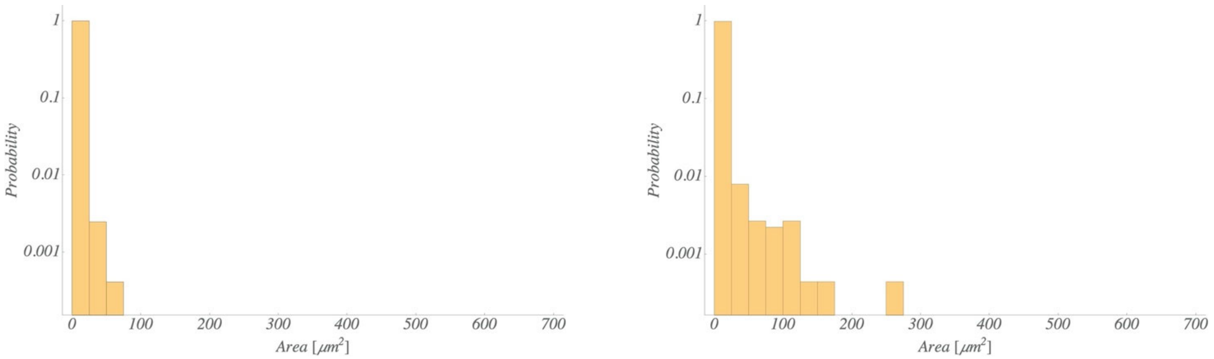

Figure 2. Histogram agglomerate size distribution (in μm²) CNF 1.0 and 1.5 wt% US.

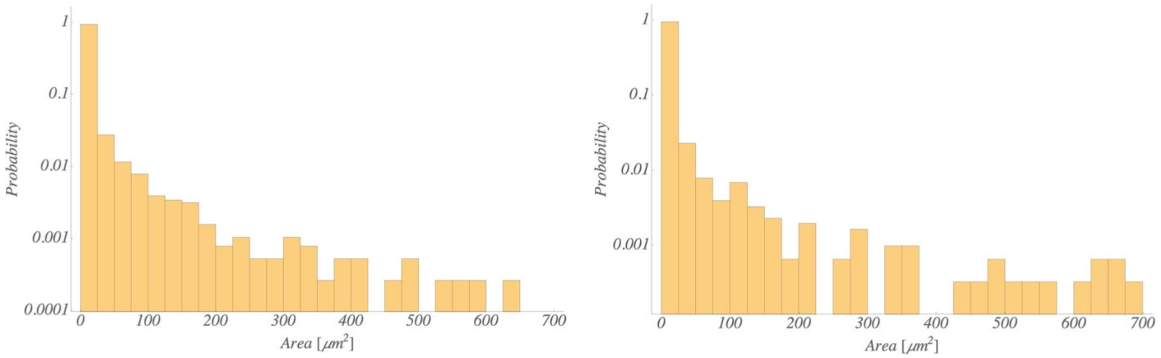

Figure S3. Histogram agglomerate size distribution (in μm²) CNT 1.0 and 1.5 wt% US.

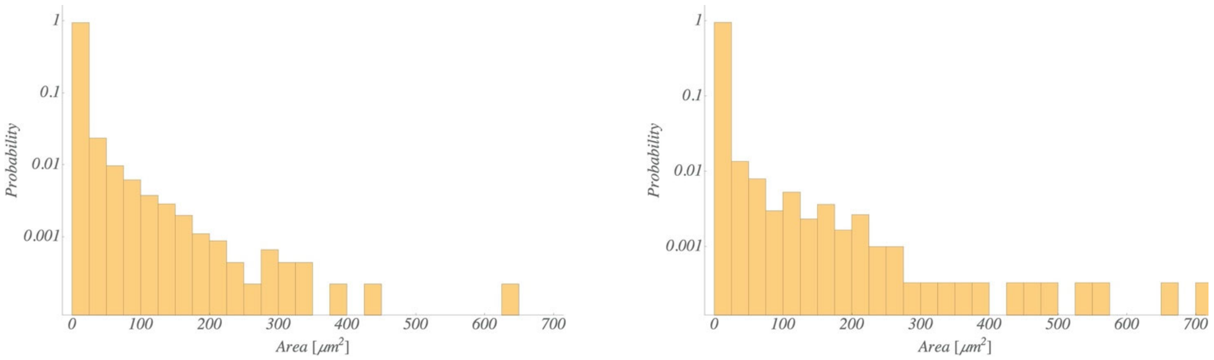

Figure S4. Histogram agglomerate size distribution (in μm²) CNToxi 1.0 and 1.5 wt% US.

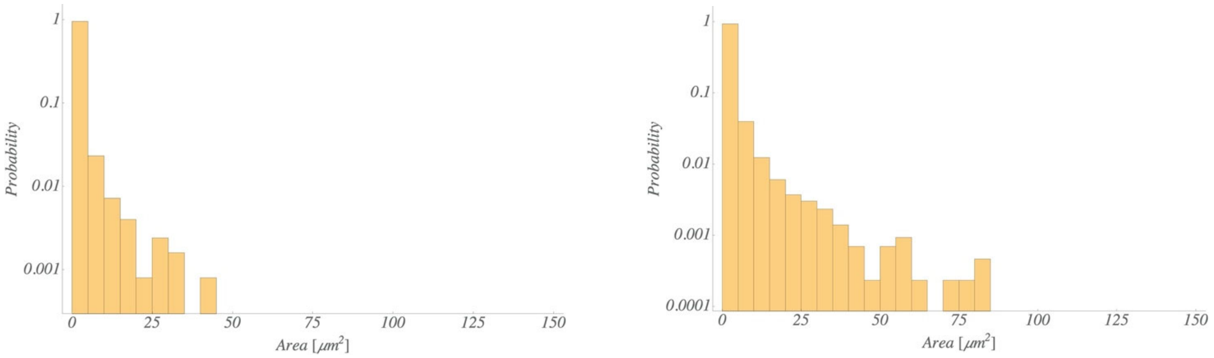

Figure S5. Histogram agglomerate size distribution (in μm²) CNF1.0 and 1.5 wt% TRM.

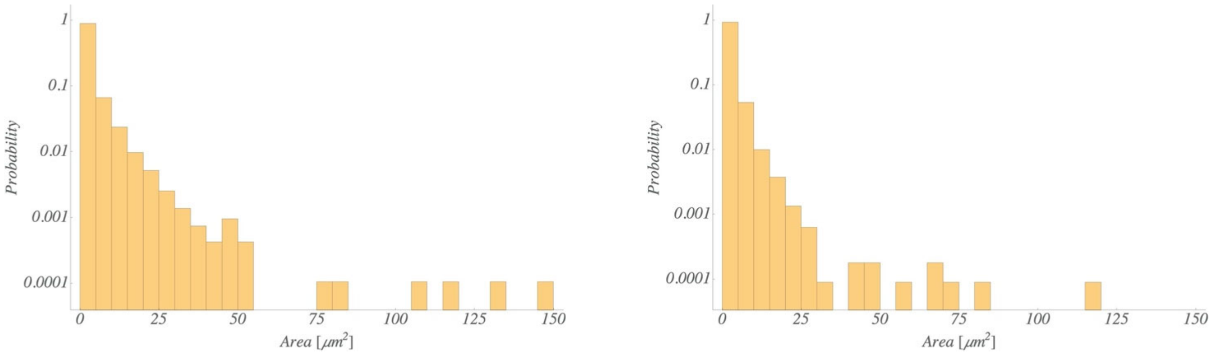

Figure S6. Histogram agglomerate size distribution (in μm²) CNT 1.0 and 1.5 wt% TRM.

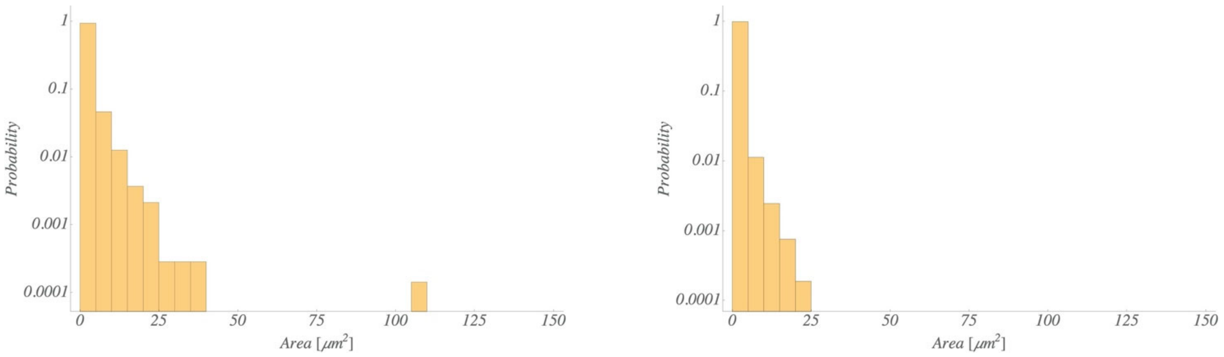

Figure S7. Histogram agglomerate size distribution (in μm²) CNToxi 1.0 and 1.5 wt% TRM.

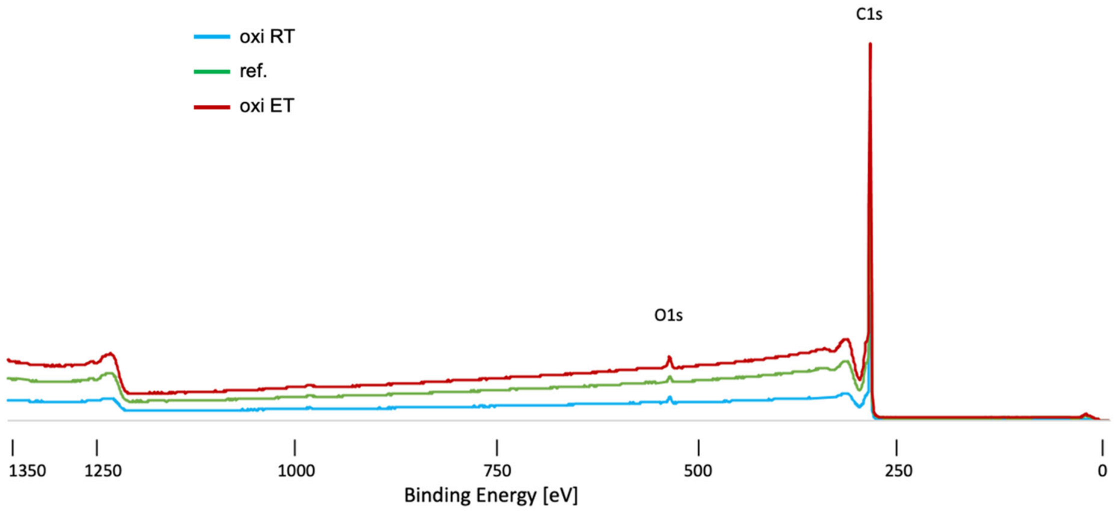

Figure S8. XPS curves of CNT oxidized at RT (oxi 21), ET (CNT oxi 120°) and a reference sample.

Table S1. XPS results of the CNT reference sample, The RT oxidized CNT and the 120° oxidized CNT.

	CNT [Atomic %]	CNT RT [Atomic %]	CNT 120°C [Atomic %]
C1s	99.9	97.6	97.3
O1s	0.1	2.4	2.7
C1s sp2	88.6	85.2	84.2

C1s sp3		0.3	0.0
C1s C-O		1.4	1.2
C1s C=O		0,6	0.8
C1s O-C=O		1.2	1.7
C1s Pi-Pi*	11.4	8.9	9.3
O1s C=O aromatic		0.6	0.6
O1s C-O		0.9	1.2
O1s =2 C=O		0.2	0.3
O1s OC=O*/C=O aliphatic		0.8	0.6

Table S2. values for G′ at 2 rad/s for different filler grades of US and TRM dispersion, evaluated through frequency sweep tests.

	**CNT**	**CNToxi**	**CNF**	**CNT**	**CNT oxi**	**CNF**
		G′ at 2 rad/s [Pa]			G′ at 2 rad/s [Pa]	
0.5 wt%	140	170	28.5	1120	80	0.16
1.0 wt%	408	623	173	1690	1000	22.3
1.5 wt%	1240	1720	409	5870	3590	80.5

Table S3. values for the damping factor at 2 rad/s for different filler grades of US and TRM dispersion.

	**CNT**	**CNToxi**	**CNF**	**CNT**	**CNT oxi**	**CNF**
		US tan(δ) at 2 rad/s			TRM tan(δ) at 2 rad/s	
0.5 wt%	0.112	0.145	0.54	0.49	1.123	98.23
1.0 wt%	0.141	0.118	0.17	0.275	0.537	2.94
1.5 wt%	0.117	0.113	0.423	0.213	0.442	1.492

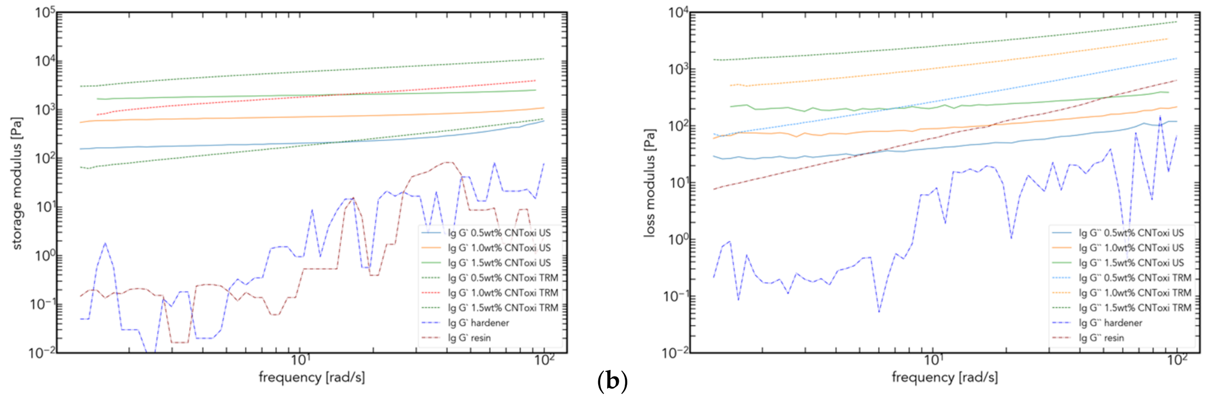

(**a**) (**b**)

Figure S9. example of frequency sweep plots for different filler grades of CNToxi for both, TRM and US: **a**) storage modulus **b**) loss modulus.

(**a**) (**b**)

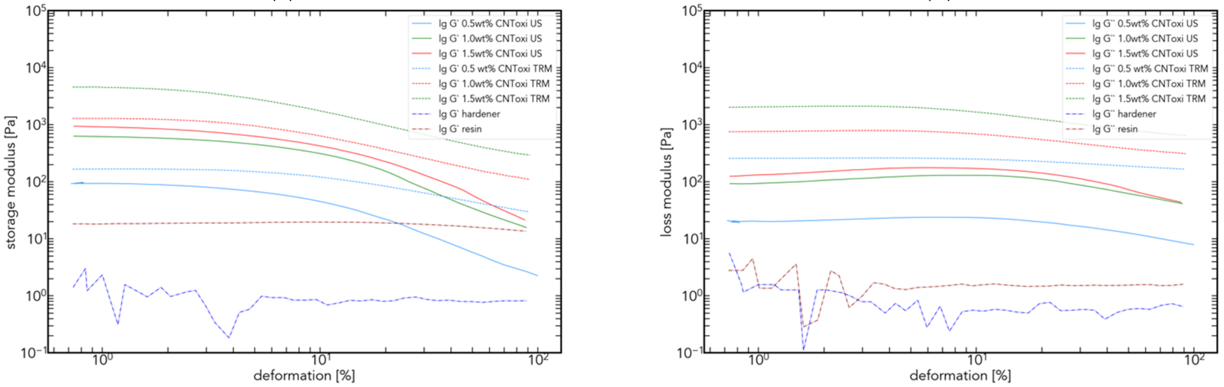

Figure S10. example of amplitude sweep plots for different filler grades of CNToxi for both, TRM and US: **a**) storage modulus **b**) loss modulus.

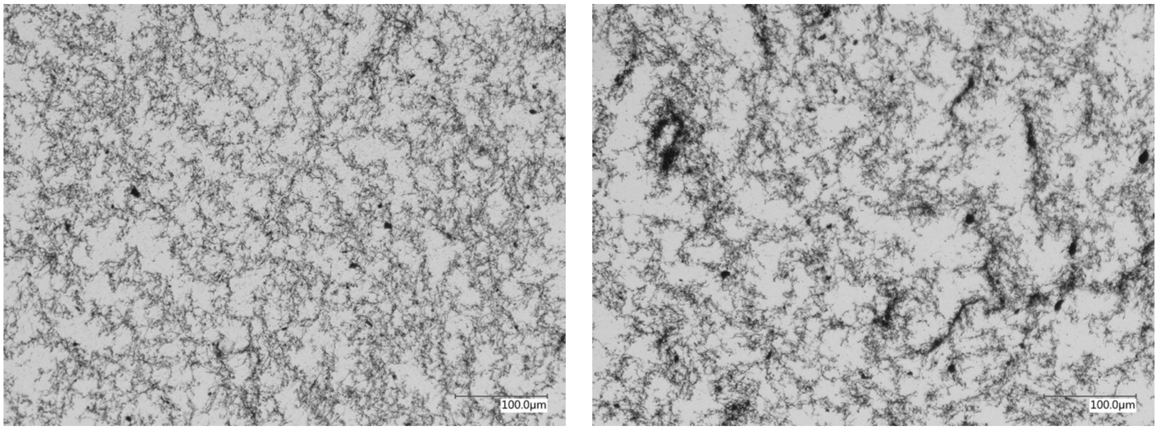

Figure S11. CNF 1.0 and 1.5 wt% US dispersion.

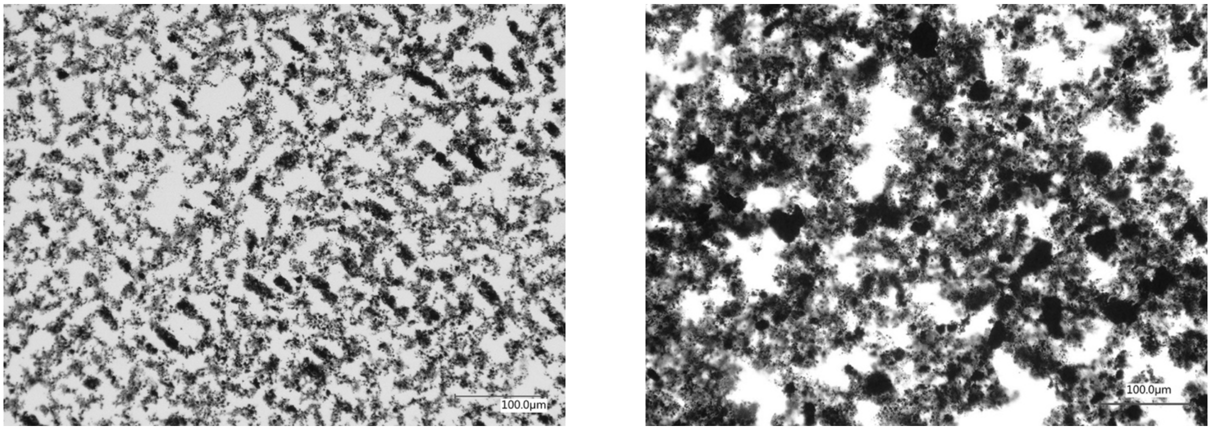

Figure S12. CNToxi 1.0 and 1.5 wt% US dispersion.

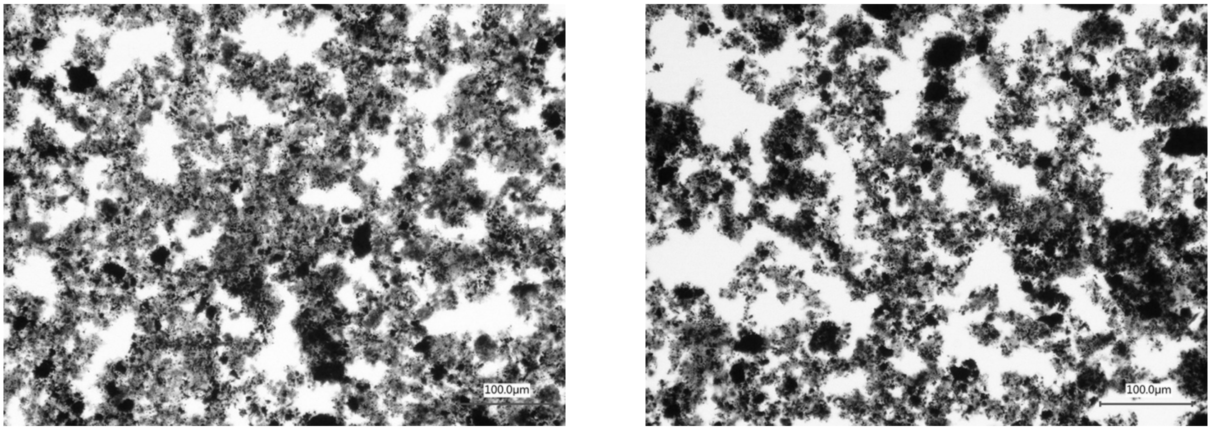

Figure S13. CNT 1.0 and 1.5 wt% US dispersion.

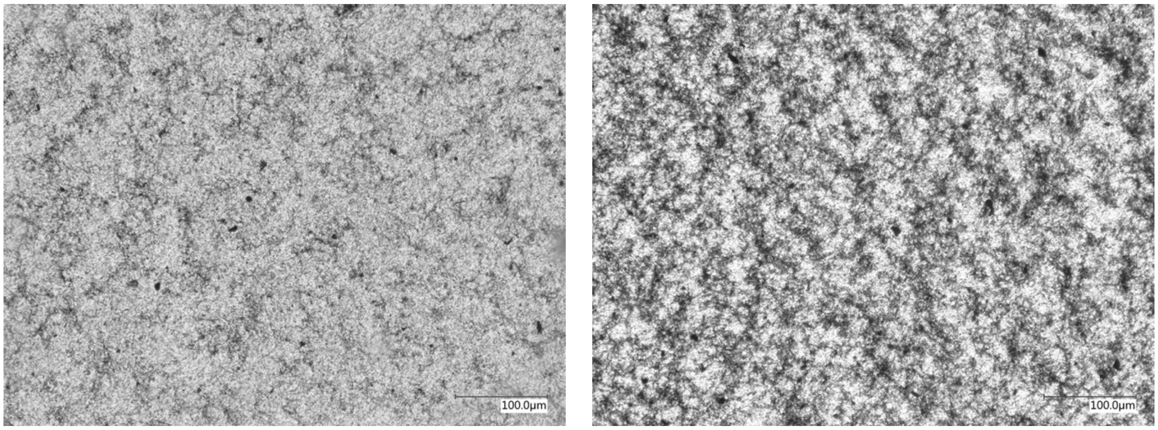

Figure S14 CNF 1.0 and 1.5 wt% TRM dispersion.

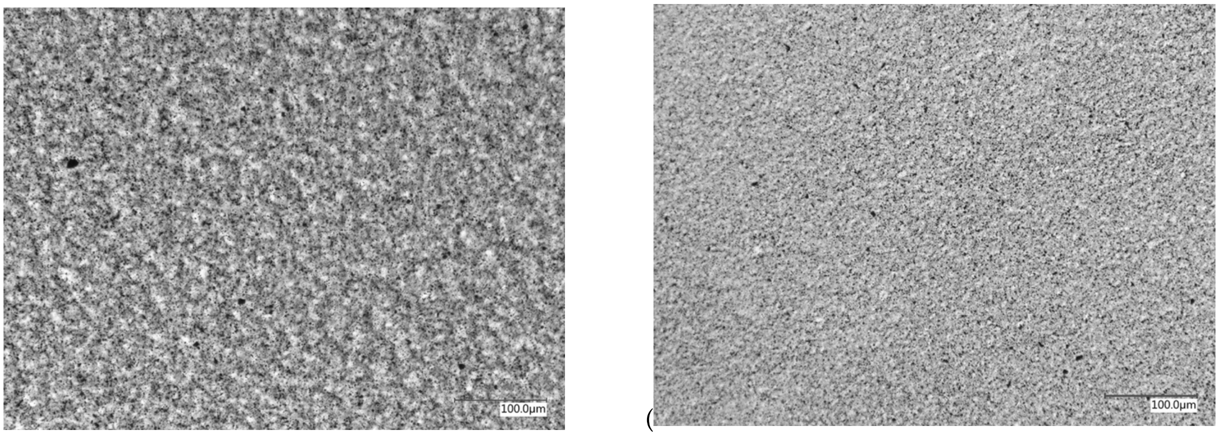

Figure S15. CNToxi 1.0 and 1.5 wt% TRM dispersion.

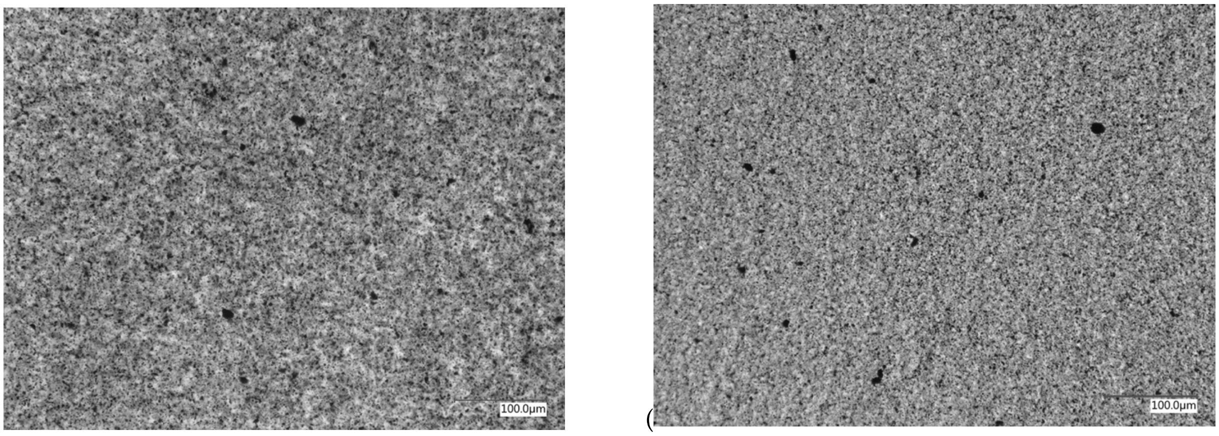

Figure S16. CNT 1.0 and 1.5 wt% TRM dispersion.

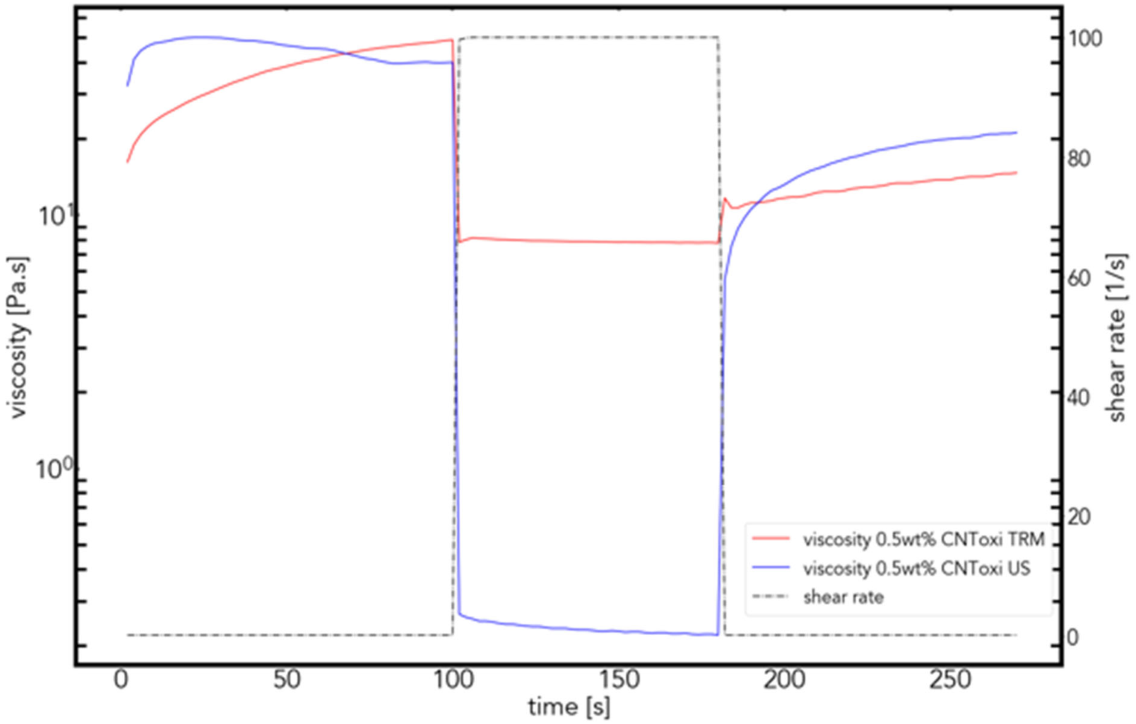

Figure S17. thixotropic behaviour evaluated for 0.5 wt% CNToxi US and TRM samples. Both samples showed the assumed thixotropic behaviour, which was more pronounced in the US sample.

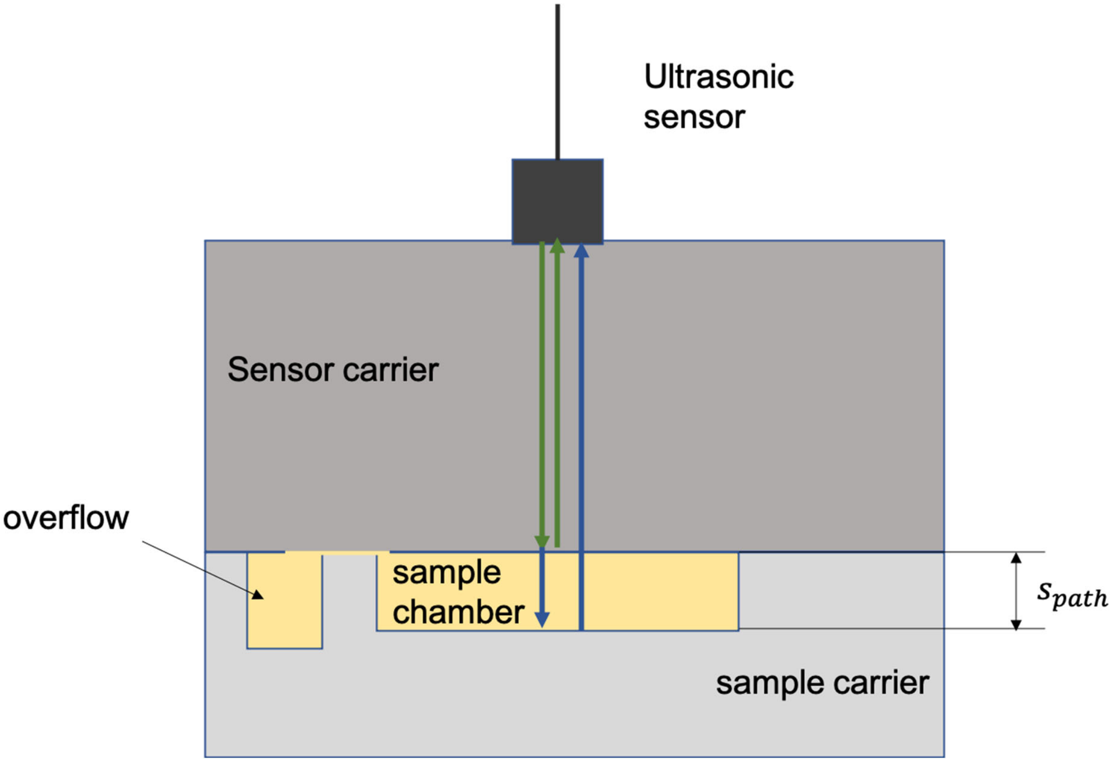

Figure S18. Setup of the felection measurement to determine the speed of sound in the used hardener matrix.

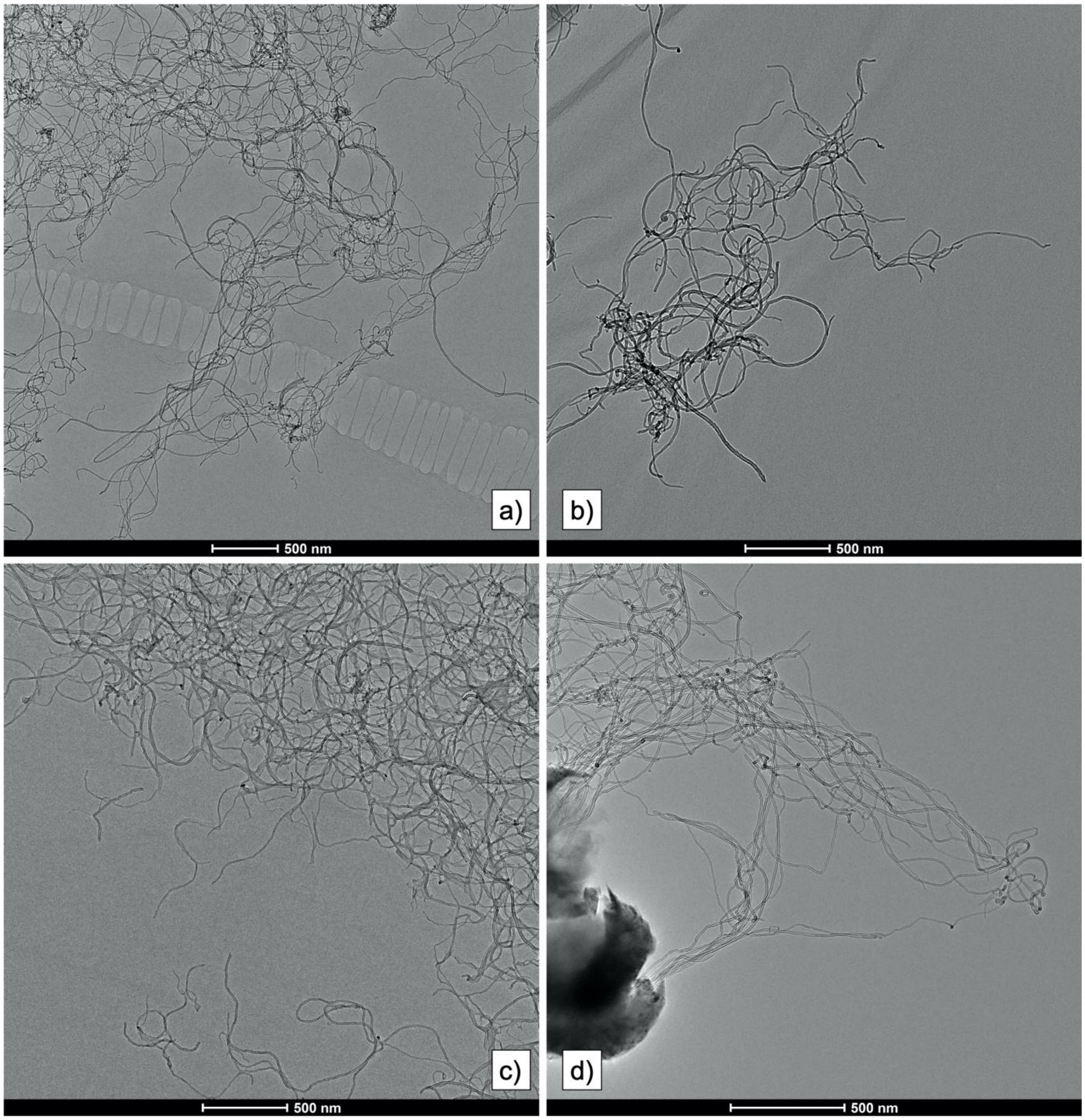

Figure S19. TEM images of **a**) CNToxi US, **b**) CNT US, **c**) CNT TRM, **d**) neat CNT to check on possible length reduction and damages through the ultrasonic dispersion process.

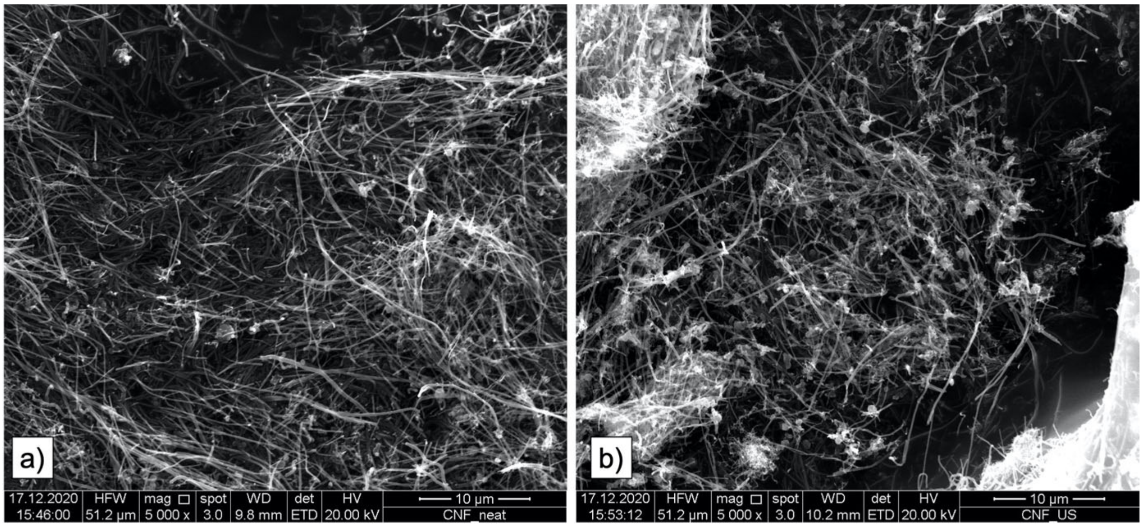

Figure S20. SEM images of **a**) neat CNF and **b**) CNF US to check on possible length reduction and damages through ultrasonication.

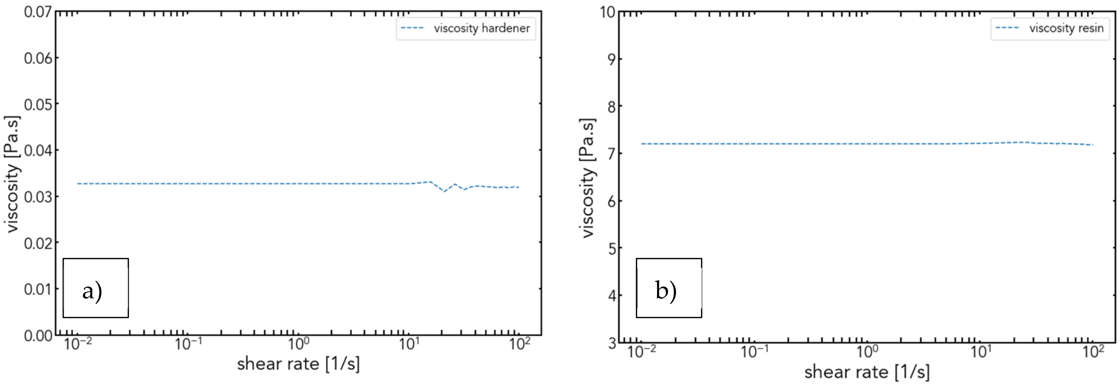

Figure S21. viscosity plots for **a**) hardener matrix and **b**) resin matrix.
